# Novelty, variability, and resilience: Exploring adaptive cycles in a marine ecosystem under pressure

**DOI:** 10.1007/s13280-025-02181-1

**Published:** 2025-04-22

**Authors:** Yosr Ammar, Riikka Puntila-Dodd, Maciej T. Tomczak, Magnus Nyström, Thorsten Blenckner

**Affiliations:** 1 Department of Environmental Research and Monitoring, Swedish Museum of Natural History, P.O. Box 50007, 104 05 Stockholm, Sweden; 2 Stockholm Resilience Centre, Stockholm University, 106 91 Stockholm, Sweden; 3 Marine and Freshwater Solutions, Finnish Environment Institute, Helsinki, Finland; 4 Department of Environmental and Marine Biology, Åbo Akademi University, Henrikinkatu 2, 20500 Turku, Finland; 5 Department of Aquatic Resources (SLU Aqua), Swedish University of Agricultural Sciences, P.O. Box 7018, 750 07 Uppsala, Sweden

**Keywords:** Adaptive cycle, Ecological network analysis, Ecosystem model and scenarios, Novelty, Reorganization, Resilience

## Abstract

Marine ecosystems are increasingly reshaped by climate change and human activities, resulting in novelty in species assemblages that have shifted beyond historical baselines. One unresolved question is how novelty influences resilience. Here, we examine how novelty arises in ecosystems when they transition through phases and affects resilience using the adaptive cycle framework. We use results from an ecosystem model of the Finnish Archipelago Sea (Baltic Sea) under contrasting climate, nutrient load and fishing scenarios. We quantify novelty in species composition and biomass and use ecological network analysis indices to identify adaptive cycle phases and resilience. Results suggest resilience decreases with higher novelty under warmer climate scenarios. Low nutrient load scenarios facilitate faster adaptive cycles and greater resilience than high nutrient load scenarios under the same climate conditions. Connecting network indices to the adaptive cycle helps to understand how the growing human-induced novelty influences resilience, supporting core resilience theory.

**Supplementary Information:**

The online version contains supplementary material available at 10.1007/s13280-025-02181-1.

## Introduction

Marine ecosystems are under unprecedented pressures from climate change and human activities, including overfishing, habitat destruction, pollution, and species introduction (McCauley et al. 2015; Jouffray et al. 2020). The ability of ecosystems to cope with perturbations depends on resilience. Ecosystem resilience describes the capacity of an ecosystem to persist disturbance, reorganize after shock and adapt to change while sustaining its major structure, functions and identity (Gunderson 2000; Folke et al. 2010).

**Fig. 1:**
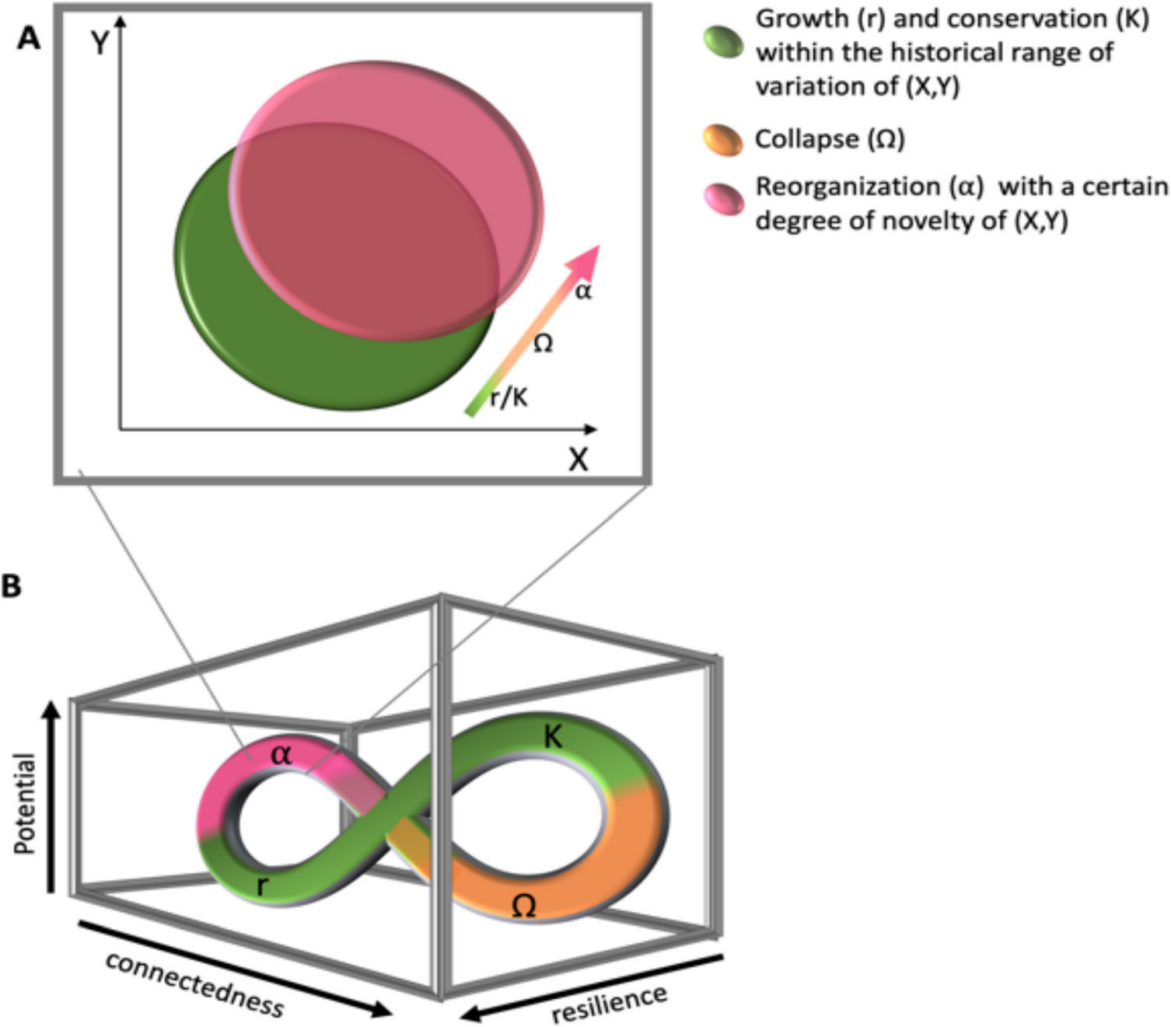
**A** Novelty is described by a shift (from the space within the green circle to the space within the pink circle) of the system over time beyond its past range of variation of (*X*, *Y*). (*X*, *Y*) is an example of a two-dimensional space of variables (e.g., temperature, salinity) or system components (e.g., abiotic, biotic or social, ecological). **B** The adaptive cycle comprises four phases: (*r*) rapid growth or exploitation, (*K*) slow growth or conservation, (Ω) collapse or release, and (*α*) reorganization (Holling 1986; Gunderson and Holling 2002). During the reorganization phase, novel configurations may emerge, which could be outside the historical range of variation of the system (*X*, *Y*) such as in **A**. **B** The three dimensions of the adaptive cycle, Connectedness (*x*-axis): the degree of connectedness among controlling variables in the system, Potential (*y*-axis): the inherent potential in the accumulated resources and the distribution of these resources will determine what options exist for the potential future, Resilience (*z*-axis): describes how vulnerable the system is to unexpected disturbances and surprises and its capacity to persist, reorganize and adapt (Gunderson and Holling 2002)

The cumulative effect of pressures has resulted in altered species distributions, composition, and interactions, leading to increasing novelty in species assemblages, i.e., assemblages shifting outside their past known range of variation (Fig. 1A, Williams and Jackson 2007). Novelty, a dynamic property of ecosystems, is defined as conditions not previously encountered within a considered spatiotemporal scale, and encompasses both biotic and abiotic elements (Ammar 2021). In species assemblages, novelty can manifest in the composition, abundance and food-web structure, while environmental novelty encompasses unprecedented combinations of environmental conditions (e.g., temperature, salinity, and nutrient availability). It has been identified across various ecosystems and timescales. For instance, in marine planktonic communities, fossil records show persistent novelty through time coupled with increased extinction, origination, and emigration (Pandolfi et al. 2020). Gradual change in seawater temperature, species introductions (intentional and unintentional), species range-shifts, and perturbations (e.g., storms and diseases) increased novelty in coral reef ecosystems (Graham et al. 2014). Future environmental novelty is projected to reshape oceans biodiversity and ecological functions, impacting 19% of fish stocks by 2050 and 59% by 2100 (Reygondeau et al. 2020).

The generation of novelty is inherent to the dynamics of complex adaptive systems (CAS),^1^ such as ecosystems, and plays a key role in their adaptive capacity (Allen and Holling 2010). However, its impact on ecological resilience can vary significantly depending on the context (Gunderson and Holling 2002). Similar to how genetic mutations influence organisms, novelty emerging from events such as invasions can either foster adaptation or be critically destructive to an ecosystem (Allen and Holling 2010). Consequently, knowing when novelty adds to or erodes resilience can be used in management to safeguard ecosystem services.

The adaptive cycle (Fig. 1B) framework describes how CAS evolve, adapt, and reorganize, emphasizing the interplay between stability, change, and resilience (Gunderson and Holling 2002). It capture the nonlinear cyclical nature of ecosystems, alternating phases of stability and instability reflecting variability, and consists of four phases (*r*, *K*, Ω, and *α*) each representing a development stage (Gunderson and Holling 2002) (e.g., succession of spring and summer blooms). The fast growth and exploitation phase (*r*) is characterized by rapid colonization (e.g., nutrient accumulated over winter allows rapid expansion of phytoplankton population spring bloom) of recently disturbed areas (e.g., temperature and light increase). Over time, this gives way to the conservation phase (*K*) with slower growth rates and intensified competition as material and energy accumulate and are stored (e.g., phytoplankton reach carrying capacity, zooplankton populations grow and compete for resources). This phase is punctuated by a sudden collapse/release phase (Ω) of previously stored energy, material, and space due to some disturbance events (e.g., temperature change, nutrient depletion and predation by higher trophic levels leading to nutrients release). The system will then move into the reorganization phase (*α*) where novelty can emerge (e.g., different combinations of species forming the summer bloom, Fig. 1B).

Besides the four development phases, the adaptive cycle captures key systemic properties, namely: *potential*, *connectedness,* and *resilience* (Fig. 1B, Gunderson and Holling 2002). The first dimension (*x*-axis) is the degree of *connectedness* among controlling variables in the system, such as ecological interactions, social networks, or economic linkages. Low connectedness allows for outward relations where the system is affected by outside variability, while high connectedness allows for inward relations where the system controls external variability (Chaffin et al. 2016). Hence, connectedness depicts the degree to which a system has control (Gunderson and Holling 2002). The second dimension (*y*-axis) is the inherent *potential* in the accumulated resources, such as biomass, nutrients, or capital. The distribution of these resources will determine what options exist for the potential futures. The third dimension (*z*-axis) is *resilience*, which continuously expands and contracts throughout the cycle (Holling 2001, Fig. 1B). This dimension describes how vulnerable the system is to unexpected disturbances and surprises and its capacity to persist, reorganize and adapt (Gunderson and Holling 2002). Hence, using the adaptive cycle provides an approach to explore the relationship between novelty (at the reorganization phase) and resilience (*z*-axis).

The adaptive cycle framework has been applied conceptually in various studies including plankton blooms in the Baltic Sea (Angeler et al. 2015), forest succession (Beier et al. 2009), freshwater recreational fisheries (Arlinghaus et al. 2017), biological invasions (Chaffin et al. 2016), and ecosystem management and governance (Garmestani et al. 2020). However, Sundstrom and Allen (2019) suggest that the adaptive cycle transcends mere metaphorical usage and constitutes a generic CAS feature. They demonstrate that many network indices derived from thermodynamics and information theory, such as ascendency (i.e., a measure of growth and development (Ulanowicz 1997); Table 1), capture different features of adaptive cycles (Sundstrom and Allen 2019). Additionally, in Castell and Schrenk (2020), network indices, including ascendency and capacity (i.e., the theoretical maximum of ascendency (Ulanowicz 1986); Table 1), were used to compute the adaptive cycle for a genome, a plant system, and the economic crisis in Europe. Their study used ascendency and capacity as respective indicators of connectedness and potential adaptive cycle dimensions (Castell and Schrenk 2020). Here, we adopt a similar methodology using network indices to identify adaptive cycle phases and understand ecosystem novelty, variability and resilience (Table 1).

**Table 1:** Description of the selected ecological network analysis indices formula and definitions for each adaptive cycle dimension. Throughput (*T*) is the total flow of energy and biomass through an ecosystem and its components over time, reflecting the ecosystem’s overall energy or resource utilization

Adaptive cycle dimension	Ecological network analysis indices	Ecological network analysis indices formula	Explanation of indicators choices
**Connectedness**: The degree of connectedness (interactions, network, linkages) among controlling variables in the system (Gunderson and Holling 2002). Low connectedness allows for outward relations where the system is affected by outside variability, while high connectedness allows for inward relations where the system controls external variability (Chaffin et al. 2016).	**Ascendency**: A measure of growth and development in the food-web where growth is the increase in the system activity or total system throughput, and development is the mutual information contained in a trophic flow (Ulanowicz 1986, 1997, 2000). It can be an indicator of connectedness in the system (Castell and Schrenk 2020)	$A = \sum\limits_{i,j} {\left( {T_{ij} } \right) \cdot \log \left( {\frac{{T_{ij} \cdot {\text{TST}}}}{{T_{j} \cdot T_{i} }}} \right)}$ With *T* the throughput and TST Total system throughput is the sum of all flows	Ascendency reflects the total system activity and mutual information within trophic flows, which together indicate how well-integrated and interdependent the components of the food-web are. Higher ascendency suggests that the system has more organized and efficient interactions among its parts, implying a higher degree of connectedness. This interconnectedness means the system has control of internal processes and response to external changes. Hence, ascendency can be used as an indicator of connectedness.
**Potential**: Sets limits to what is possible and the inherent potential in the accumulated resources, to determine the number of alternative options that exist for the future (Gunderson and Holling 2002).	**Capacity** or the development capacity measures the ecosystem development potential (Shannon et al. 2009) and also represents the theoretical maximum of ascendency (Ulanowicz 1986). This means that when the development capacity is at its highest, the ecosystem is as mature as it can be. It can be an indicator of potential in the system (Castell and Schrenk 2020)	$C = \sum\limits_{ij} {T_{ij} \log \left( {\frac{{T_{ij} }}{{{\text{TST}}}}} \right)}$ With *T* the throughput and TST Total system throughput is the sum of all flows	Capacity measures the food-web maximum development and organizational complexity, reflecting its ability to sustain complex processes and structures. When the development capacity is at its highest, it indicates that the system is fully mature and has reached its maximum organizational complexity and efficiency. This high capacity sets the limits of what is possible within the ecosystem, determining the range of alternative options and pathways it can take in the future. Thus, capacity reflects the inherent potential of the system and existing future potential options.
**Resilience**: The capacity of an ecosystem to persist, reorganize, and adapt to change while sustaining its major functions and identity (Gunderson 2000; Folke et al. 2010). It determines how vulnerable the system is to unexpected disturbances and surprises that can exceed or break that control (Gunderson and Holling 2002).	**Overhead flow**: Is the product of the unconstrained component of Shannon’s flow diversity of energy/material within a food-web and the Total System Throughput (TST) and can be explained as functional redundancy (Ulanowicz 2018). It indicates the change in degrees of freedom of the system (Ulanowicz 1986, 2004; Heymans et al. 2007) or the distribution of energy flow pathways in the system (Heymans et al. 2014). This could indicate the vulnerability of the system to unexpected events. It can be an indicator of systems resilience (Heymans et al. 2007).	$R = - \sum\limits_{i = 1}^{n} {\sum\limits_{j = 1}^{n} {(T_{ij} ) \cdot \log \left( {\frac{{T_{ij}^{2} }}{{\sum\nolimits_{j = 1}^{n} {T_{ij} \cdot } \sum\nolimits_{i = 1}^{n} {T_{ij} } }}} \right)} }$ With *T* the throughput and TST Total system throughput is the sum of all flows	Overhead flow represents the functional redundancy and diversity of energy or material flow within the food-web. High overhead flow indicates a greater number of alternative pathways and flexibility in energy distribution, which allows the system to absorb and adapt to disturbances without collapsing. This redundancy and distribution of pathways provide a buffer against unexpected events, enhancing the system’s ability to recover, reorganize, and maintain functions despite disruptions. Therefore, overhead flow can serve as an indicator of the system’s resilience and its capacity to withstand, cope, reorganize, and adapt to environmental changes and shocks

In this paper, we explore how novelty emerges in the Finnish Archipelago Sea (FAS) ecosystem, Northern Baltic Sea, and examine what role it plays for resilience. Using the adaptive cycle framework, we analyze ecosystem transitions between phases of growth, collapse and reorganization in response to disturbances. We ask: how do climate change and anthropogenic pressures drive the emergence of novelty and ecosystem changes that influence resilience? Through an ecosystem model of the FAS with contrasting future climate change, nutrient load, and fishing scenarios, we first quantify future novelty in species assemblages (biomass and composition). Second, we combine these results with network indices extracted from the ecosystem model to assess ecosystem resilience under above-mentioned pressures and identify the phases of the adaptive cycle. This process allows examining how novelty emerges in response to combined pressures and provides insights into ecosystem resilience across scenarios.

## Materials and methods

### Study area—Finnish Archipelago Sea

The Baltic Sea is one of the world’s most impacted semi-enclosed seas due to climate change, fishing, and pollution. Environmental novelty in the Baltic has fluctuated nonlinearly over three decades due to rising temperatures and declining salinity, with projections suggesting impacts on food-web structure and functions (Ammar et al. 2021b; Blenckner et al. 2021).

**Fig. 2:**
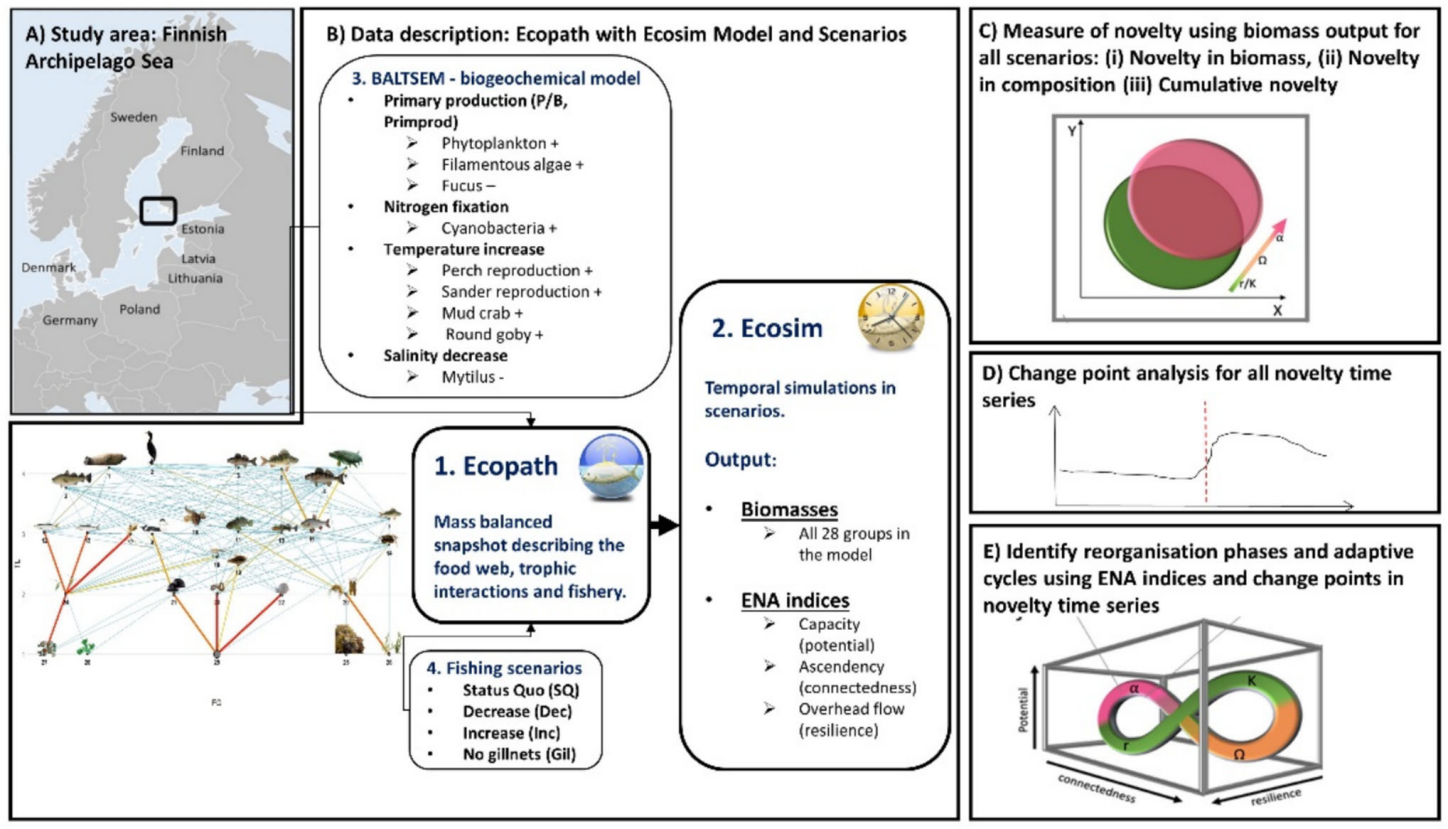
The methodology is illustrated in five steps: **A** Study area: Location of the Finnish Archipelago Sea in the Baltic Sea. **B** Data description: Ecopath with Ecosim model and scenarios. Building the model is not within the scope of this paper and detailed description can be found in Puntila-Dodd et al. (2022) and Uusitalo et al. (2023). Detailed scale of the food-web is found in SupInfo Figs. S1, S2. **C** Measuring novelty metrics using three methods in all scenario combinations described in Table 2. **D** Identifying change points in mean in all novelty time series. **E** Using the change points of novelty time series and the ecological network analysis (ENA) indices, we identify the reorganization phase. The following phases of the adaptive cycle are identified based on the description in Gunderson and Holling (2002). The ecological network analysis indices formula and explanations are found in Table 1

The FAS located at the entrance of the Northern Baltic Sea (Fig. 2A) holds significant social-ecological importance. It serves as a nursery area for fish and birds (Virtanen et al. 2018), supports a rich cultural heritage, and sustains socioeconomic activities such as fisheries and tourism. However, it is exposed to the same pressures as the wider Baltic region. Notably, agricultural nutrient runoff contributes to recurring bottom anoxia and algal blooms (HELCOM 2020).

The FAS has also suffered from the introduction of invasive species, impacting the ecosystem structure and functions. For example, the fish-hook water flea (*Cercopagis pengoi*, Ostroumov) was detected in the Northern Baltic Sea in the early 1990s, and has since increased in abundance (Antsulevich and Välipakka 2000), competing with fish larvae and other zooplanktivores (e.g., mysids) for food (Lehtiniemi and Lindén 2006). The flea has also become a frequent prey item for herring (*Clupea harengus*) (Gorokhova et al. 2005). In the 2000s, the round goby (*Neogobius melanostomus*) and Harris mud crab (*Rhithropanopeus harrisii*) invaded the FAS, demonstrating a remarkable adaptive capacity to local prey, increasing abundance, and becoming part of the native predators prey field (Puntila et al. 2019).

Actions to improve the water quality of the Baltic Sea, including FAS, have been implemented at local, regional, and European levels (Elmgren et al. 2015). Notably, the regional Baltic Sea Action Plan (BSAP, HELCOM 2007) is a science-based adaptive management plan aligned with EU directives (e.g., Marine Strategy Framework Directive) to achieve Good Environmental Status.

### Data: Ecosystem model and scenarios

#### Ecopath with Ecosim

Ecopath with Ecosim^2^ is a widely used tool for modeling aquatic food-web interactions. It is built around three main components: Ecopath—a static, mass-balanced snapshot of the system; Ecosim—a time dynamic simulation module; and Ecospace—a spatial and temporal dynamic module (Christensen et al. 2008). The latter is not applied in our case. A comprehensive description of the FAS model can be found in Puntila-Dodd et al. (2022) and Uusitalo et al. (2023). We provide a summary of the model and scenarios in this study (Fig. 2B).

The mass-balanced FAS ecosystem model comprises 28 biotic groups ranging from primary producers to fish, seals, and birds (Fig. 2B, Fig. S1). The static Ecopath model was initially developed using the year 2000 data, and Ecosim was calibrated with 18 years of monitoring data (2001–2018; Fig. 2B1–2). The model includes two life stages for sander (*Sander lucioperca*) and perch (*Perca fluviatilis*), both important species for the coastal fishery, and three invasive species–round goby, Harris mud crab, and fish-hook water flea. The non-indigenous species invasion process was modeled by using artificial fishing fleets controlling these species until the invasions took place (sensu Langseth et al. 2012). Seven fishing fleets described fishing (and hunting): nets, traps, lines, trawls, recreational fishing, hunting, and others. Fishing in the model is effort-driven, meaning that management scenarios are implemented by altering effort in the assigned fleets. Data for fishery catches and commercial fishery efforts were obtained from the Natural Resources Institute in Finland. Cod (*Gadus morhua*) and sprat (*Sprattus sprattus*) biomasses used forcing from the output of the Central Baltic Sea model (Bauer et al. 2018) as these species do not reproduce in the FAS area. The forcing term modifies the production of these groups in the model. The coastal model is not nested within the broad Baltic Sea model and does not incorporate two-way interactions with the Central Baltic Sea model.

Simulated time series describing environmental conditions and primary production changes for nutrient and climate scenarios were received from the biogeochemical model BALTSEM (BAltic sea Long-Term large Scale Eutrophication Model, (Gustafsson et al. 2012; Fig. 2B3). Before model calibration, the initial conditions were evaluated against basic assumptions following the criteria by Link (2010). After calibrating from 2000 to 2018, climate, nutrient load, and fishing scenarios were used for simulations from 2019 to 2090.

A Monte Carlo routine was applied to estimate the uncertainty range of the results. This was executed using an Ecosampler routine by resampling basic Ecopath parameters (Steenbeek et al. 2018). The routine is used for analyzing the robustness of the model’s results and visualizing the uncertainty (details in the SupInfo, Steenbeek et al. 2018). All projections were compared to a retrospective baseline run (2000–2018).

#### Scenarios: Climate change, nutrient load, and fishing management

A total of 16 scenarios were applied in our case study, combining four fishing management scenarios with two nutrient load scenarios and two climate projections (Table 2). The fisheries management scenarios were developed based on expert opinion to illustrate the potential impacts of changes in fisheries: Status Quo (SQ) assumes a continuation of the current fishing effort; The decreasing fishing scenario (Dec) describes the decrease in fishing effort in all fleets by 50% compared to the SQ scenario; The increasing fishing scenario (Inc) assumes an increase in the fishing effort by 100% compared to the SQ scenario; The fourth scenario (Gil) assumes the cessation of professional gillnet fishery. The biogeochemical scenarios obtained from the BALTSEM model include nutrient load scenarios in the reference (REF) scenario (see Saraiva et al. 2019) and the nutrient load outlined in the BSAP (HELCOM 2007). Two climate scenarios are used for our simulations, i.e., the Representative Concentration Pathways RCP4.5 and RCP8.5 (IPCC 2014).

**Table 2:** Combination of scenarios built for the Finnish Archipelago Sea Ecopath with Ecosim scenarios. Only combinations of climate and nutrient load scenarios with Status Quo (SQ) are presented in the main manuscript

Climate scenarios	Nutrient load scenarios	Fishing scenarios
Climate representative concentration pathways RCP4.5 (IPCC 2014)	Baltic sea action plan BSAP (HELCOM 2007)	Status quo (SQ)
		Decrease in fishing effort by 50% (Dec)
		Increase in fishing effort by 100% (Inc)
		No gillnets fishery (Gil)
	Reference scenario (REF) described in Saraiva et al. (2019)	Status quo (SQ)
		Decrease in fishing effort by 50% (Dec)
		Increase in fishing effort by 100% (Inc)
		No gillnets fishery (Gil)
Climate representative concentration pathways RCP8.5 (IPCC 2014)	Baltic sea action plan BSAP (HELCOM 2007)	Status quo (SQ)
		Decrease in fishing effort by 50% (Dec)
		Increase in fishing effort by 100% (Inc)
		No gillnets fishery (Gil)
	Reference scenario (REF) described in Saraiva et al. (2019)	Status quo (SQ)
		Decrease in fishing effort by 50% (Dec)
		Increase in fishing effort by 100% (Inc)
		No gillnets fishery (Gil)

Considering fishing pressure had a relatively minor impact on ecosystem structure and resilience compared to climate and nutrient load scenarios, we choose to present the results in the main manuscript with the SQ fishing scenario. Other fishing scenarios results are provided in Figs. S9–S12.

#### Ecological network analysis indices

Different environmental indices, such as those extracted from ecological network analysis, can be used as a measurement for the ecosystem state (e.g., Ulanowicz 1986). This network analysis applies a set of algorithms to evaluate the flows of energy and material within ecosystems from which a suite of systems properties can be derived. The analysis enables the representation of the interactions among species and functional groups in the food-web and provides insights into ecosystem maturity, stability and resilience (Shannon et al. 2009; Tomczak et al. 2013; Heymans et al. 2014).

In this study, we use the ecological network analysis tool within Ecopath to extract capacity, ascendency, and overhead flow (see descriptions in Table 1, Fig. 2E). We consider the network indices ascendency and capacity as respective indicators of connectedness and potential (Castell and Schrenk 2020). We use overhead flow*,* which describes degrees of freedom in energy flow pathways, as an indicator of resilience (Ulanowicz 1986, 2004; Heymans et al. 2007, 2014). These indices are later used to identify adaptive cycles.

### Ecosystem overview under compound pressures

**Fig. 3:**
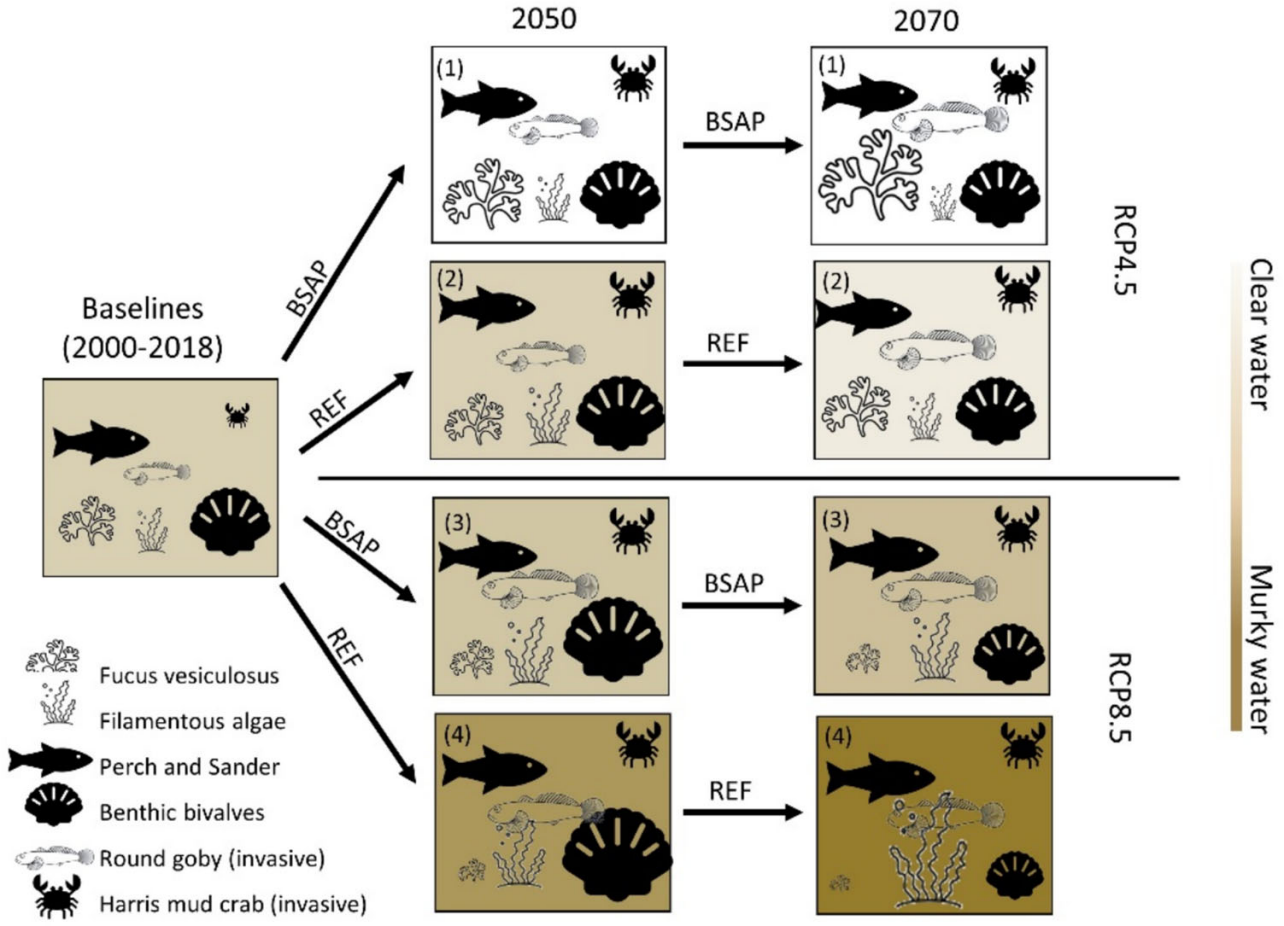
Schematic ecosystem archetypes of the trajectories of ecosystem characteristics from the baseline (2000–2018) to 2070 given for combinations of two climate projections (RCP4.5 and RCP8.5), two nutrient loads scenarios (Baltic Sea Action Plan BSAP, and Reference REF) and one fishing (Status Quo SQ) scenario. Key species and water clarity (background color) for each trajectory are indicated. The size of the species icon illustrates the biomasses. The number on the top left in each box represents different identified trajectories. Combinations of climate, nutrient load, and other fishing scenarios, as well as specific food-web biomasses and flows for years 2050, 2070, and 2090, are illustrated in the SupInfo (Fig. S4–S8)

To illustrate key differences between scenarios, we developed visual representations of ecosystem archetypes (Fig. 3) based on the ecosystem state, biomass and flows (Figs. S5–S8) for the baseline (2000–2018) and three future years (2050, 2070, and 2090). These scenario outcomes are not static, but represent possible future pathways of how the FAS ecosystem might evolve under different pressures. We will refer to them as “trajectories” in the following.

The FAS is a bottom-up-driven ecosystem. Thus, we selected three characteristics that describe the main primary production components: the abundance of the macroalgae *Fucus vesiculosus* (hereafter *Fucus*), the abundance of filamentous algae, and water transparency. All three are indicators of the ecosystem’s environmental status aligned with the EU Marine Strategy Framework Directive (Korpinen et al. 2022). *Fucus* is a habitat-forming species, which depends on the water transparency and salinity (Rinne and Salovius-Laurén 2020). This species is in a poor state during baseline years (Fig. S5). Annual filamentous algae, in turn, has benefitted from eutrophication, negatively affecting *Fucus* (Isæus et al. 2004). We, moreover, include benthic bivalves to represent the benthos, the mud crab and round goby to show the state of the invasions, and the native fish species perch and sander to indicate valuable fishing opportunities.

### Novelty and change-point analyses

Novelty is commonly measured as the minimum dissimilarity compared to a specific baseline (Fig. 1A; Radeloff et al. 2015). We calculate novelty over time as:

$${\text{Novelty}} = {\text{min}}\,D_{{{\text{target,}}\,\,{\text{baseline}}}}$$

where *D* is dissimilarity, baseline is the calibration period (2000–2018), and targets include the years between 2019 and 2090. We compute the distance to the closest food-web configuration in the baseline period considering all model uncertainty range, for all scenarios time series.

We calculate novelty using three dissimilarity measures to capture changes in the food-web (Fig. 2C): (i) Novelty in biomass: dissimilarity of species biomasses for each year to the closest species biomasses in the baseline period using the Euclidean distance; (ii) Novelty in composition: the turnover in species composition (proportion of species) in the whole food-web for each year compared to the baseline using the Hellinger distance, which lower the importance of the most abundant species (in this case primary production) (Rao 1995); (iii) Novelty in individual species (also termed as cumulative novelty): the sum of biomass differences compared to the baseline for each species. This produces time series for each novelty measure representing the degree of novelty over time relative to the baseline. We accounted for uncertainty by performing calculations across the full uncertainty range of all 16 scenario combinations, averaging results within these ranges, and deriving a single novelty time series per novelty measure (total of 48 time series).

Change-point analysis detects statistically significant shifts in a time series, indicating periods of substantial change. In this study, we use this analysis to detect significant shifts in the novelty time series (Fig. 2D), to identify when the ecosystem undergoes substantial changes, e.g., structural and functional changes. To do this, we apply the *envcpt* function from the *EnvCpt* package (Killick et al. 2021), which detects change points by fitting up to 12 different models that account for variations in trend, mean, and autocorrelation. For this study, we focused specifically on detecting “change-points in mean” identifying significant shifts in the mean values of the novelty time series, regardless of the direction of change. The minimum segment length was set to 10 years. This analytical approach was applied consistently to all novelty time series.

These analyses were performed using R language and environment (R Core Team 2022).

### Identifying adaptive cycle phases

We visualize novelty and resilience (overhead flow) time series against each other to assess potential co-variation over time. To understand how the FAS ecosystem transitions between adaptive cycle phases, we quantitatively identify a number of reorganization phases/events and qualitatively classify the remaining phases.

The three network indices, ascendency, capacity and overhead flow, respective indicators of connectedness, potential and resilience, were smoothed by applying the *geom_smooth* function (in *R*) to improve visualization. First, we identify the reorganization phase by placing the previously detected change points from the novelty time series onto the three network indices in each scenario. Using these change points and the expected patterns described in the adaptive cycle framework (Gunderson and Holling 2002), a downward trend in connectedness and an upward trend in potential and high resilience (Fig. 1B; Table S1), we determine when the system enters the reorganization phase, where novelty is expected to emerge. The remaining phases were qualitatively classified based on the trends described in the adaptive cycle framework. The collapse phase precedes reorganization, showing downward trends in connectedness and potential and an upward trend in resilience. The fast (*r*) and slow growth (*K*) phases follow reorganization with upward trends in connectedness and potential and downward trends in resilience. Given the difficulty in distinguishing between the *r* and *K* phases based on network indices trends, we group them into a single growth (*r*/*K*) phase.

The phases show how frequently and how fast/slow the ecosystem reorganized under varying pressures. The number of cycles does not necessarily indicate changes in resilience. However, reorganization can lead to a new or different trajectory, where resilience could increase or decrease depending on the resulting structure. As our model does not include extreme events, we assume the order of the phases remains unchanged.

## Results

First, we provide a summary describing the trajectories of the FAS ecosystem generated under various management scenarios. Next, we present the results by trajectory for (i) novelty in biomass and composition over time, including significant change points, (ii) the consequences of novelty for ecosystem resilience, and (iii) the reorganization phases and the adaptive cycles under the different scenarios.

### Model trajectories description

The ecosystem baseline (2000 to 2018), to which future ecosystem states are compared, is characterized by eutrophic conditions with low *Fucus* biomass, low water transparency, high filamentous algae biomass, and medium to low invasive species biomass. Four distinct future ecosystem trajectories evolve from the baseline under combined climate, nutrient load, and SQ fishing pressure scenarios (Fig. 3, Figs. S4–S8).

In the nutrient reduction scenario (BSAP) combined with the RCP4.5 climate scenario, a clear water state emerges (trajectory 1, Fig. 3), characterized by increased water transparency and the following increase in *Fucus* biomass (by 360% in 2070 compared to the baseline). Trajectories 2 and 3 initially experience similar eutrophic conditions to the baseline until the 2040s. Under the combination of moderate warming (RCP4.5) and current nutrient loads (REF) in trajectory 2, water quality improves slightly in 2070, which results in a little improvement of *Fucus* biomass. However, under the warmer climate (RCP8.5), named trajectory 3, the FAS stays in the same eutrophic baseline conditions despite the nutrient reduction (BSAP) scenario. In the latter scenario, *Fucus* biomass declines slowly (by 15% in 2070 compared to the baseline), while filamentous algae increase (by 124% in 2070 compared to the baseline). The scenario with the current nutrient load (REF) combined with intense sea temperature warming (RCP8.5, up to 1.8 degrees in 2070) in trajectory 4 is characterized by intense eutrophication with increased filamentous algal growth (by 179% in 2070 compared to the baseline) and a poor *Fucus* state. This highlights the complex interactions between climate and nutrient load management.

Important changes in other species’ biomasses occur. For instance, benthic bivalves biomass, which feed mainly on phytoplankton and detritus, grows faster toward 2050 but decreases toward 2070 in the RCP8.5 scenarios (trajectories 2 and 4) while remaining stable in the RCP4.5 scenarios (trajectories 1 and 3). Additionally, invasive species biomasses are different between scenarios. Round goby biomass reaches carrying capacity faster in warmer climate scenarios (trajectories 2 and 4), whereas the mud crab biomass reaches carrying capacity in all scenarios by 2050. The two native fish species, perch and sander, benefit from warmer climates (Heikinheimo et al. 2014; Kokkonen et al. 2019) and maintain their biomasses in all trajectories with the SQ fishing pressure. Further details of the resulting trajectories can be found in SupInfo.

### Emergence of novelty in the ecosystem

**Fig. 4:**
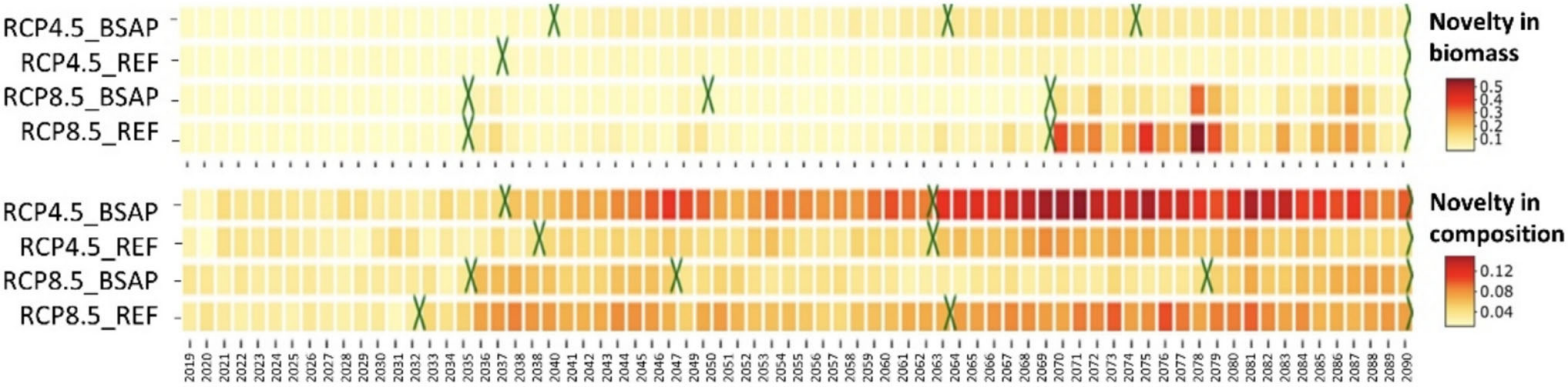
Novelty in biomass and in composition, compared to the 2000–2018 baseline for the four different trajectories. The detection of change points in the mean novelty is marked in green (*X*). Clear water trajectory 1 (RCP4.5_BSAP), intermediate trajectories 2 (RCP4.5_REF), and 3 (RCP8.5_BSAP), and the most eutrophic trajectory 4 (RCP8.5_REF). All trajectories are under the SQ fishing pressure (See SupInfo Fig. S9 for other fishing pressure scenarios)

The highest novelty in biomass was calculated for trajectory 4 (RCP8.5_REF), which is associated with the decrease in biomass in most species (e.g., *Fucus* and bivalves) and the increase in filamentous algae and heat-opportunistic species (e.g., sander and perch). Significant change points were detected in years 2035 and 2069 (Fig. 4). The second-highest novelty in biomass was calculated for trajectory 3 (RCP8.5_BSAP). Significant change points were detected in 2035, 2069, and 2049. For the clear water trajectory 1 (RCP4.5_BSAP), novelty in biomass emerges less strongly from 2040 onward compared to the baseline. Three significant change points were detected in years 2040, 2063, and 2074. The lowest novelty in biomass was calculated for trajectory 2 (RCP4.5_REF), indicating the closest biomass of species to the baseline. Only one change point was detected in year 2037.

Novelty in species composition was consistently highest in the clear water trajectory 1, which is due to the increase in the proportion of *Fucus* compared to other species in the ecosystem. Significant change points were detected in years 2037 and 2062. The most eutrophic trajectory 4 (RCP8.5_REF) shows the second-highest novelty with fluctuations over time and significant change points in 2032 and 2063 (Fig. 4). Both intermediate trajectories 2 and 3 exhibited the lowest novelty in composition, indicating similar species composition to the baseline conditions. However, they showed distinct significant change points over time: 2039 and 2062 under RCP4.5_REF, and 2036, 2047, and 2078 under RCP8.5_BSAP.

### Novelty and resilience

**Fig. 5:**
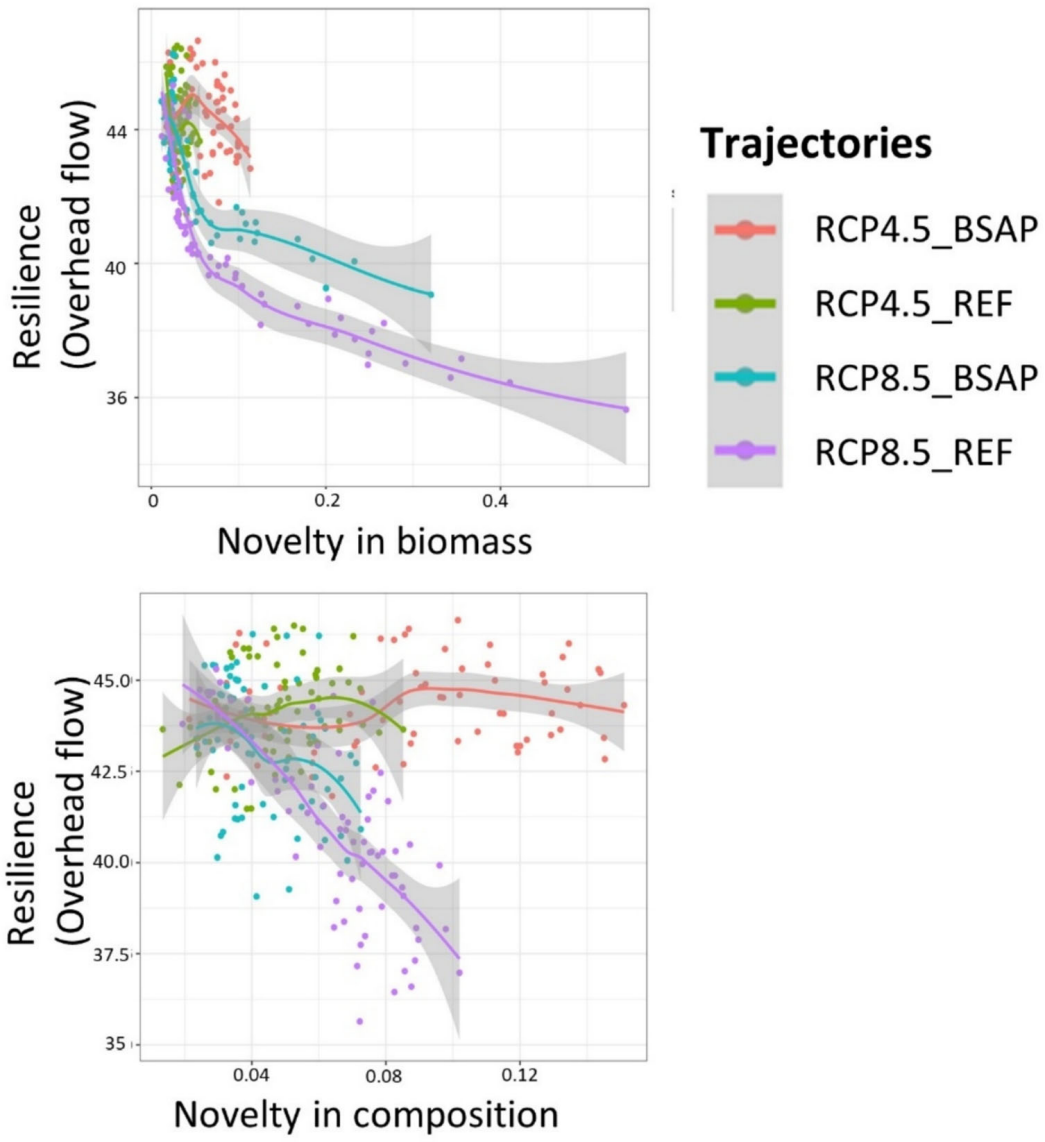
Ecosystem trajectory-specific novelty-resilience (overhead flow) relationships based on species novelty in biomass (top panels) and novelty in composition (bottom panel) for the four different ecosystem trajectories. Clear water trajectory 1 (RCP4.5_BSAP), intermediate trajectories 2 (RCP4.5_REF), and 3 (RCP8.5_BSAP) and the most eutrophic trajectory 4 (RCP8.5_REF). All trajectories are under the SQ fishing pressure (See SupInfo Figs. S11, S12 for other fishing pressure scenarios)

The results show clear differences between trajectories in resilience indicator (overhead flow) and novelty (Fig. 5). In the most eutrophic trajectory 4 (RCP8.5_REF), which has the highest novelty in biomass and the second-highest novelty in composition (Fig. 4), resilience declines abruptly with rising novelty. A similar but weaker relationship was found for trajectory 3 (RCP8.5_BSAP).

In contrast, trajectory 1 (RCP4.5_BSAP) and trajectory 2 (RCP 4.5_REF) showed no change in resilience with the increase in novelty, despite the high novelty in composition in trajectory 1. In both trajectories, novelty in biomass remained low.

Overall, resilience decreased with higher- and faster-emerging novelty in biomass but not necessarily with higher novelty in composition, particularly under warmer climate conditions.

### Dynamics of reorganization and adaptive cycles

**Fig. 6:**
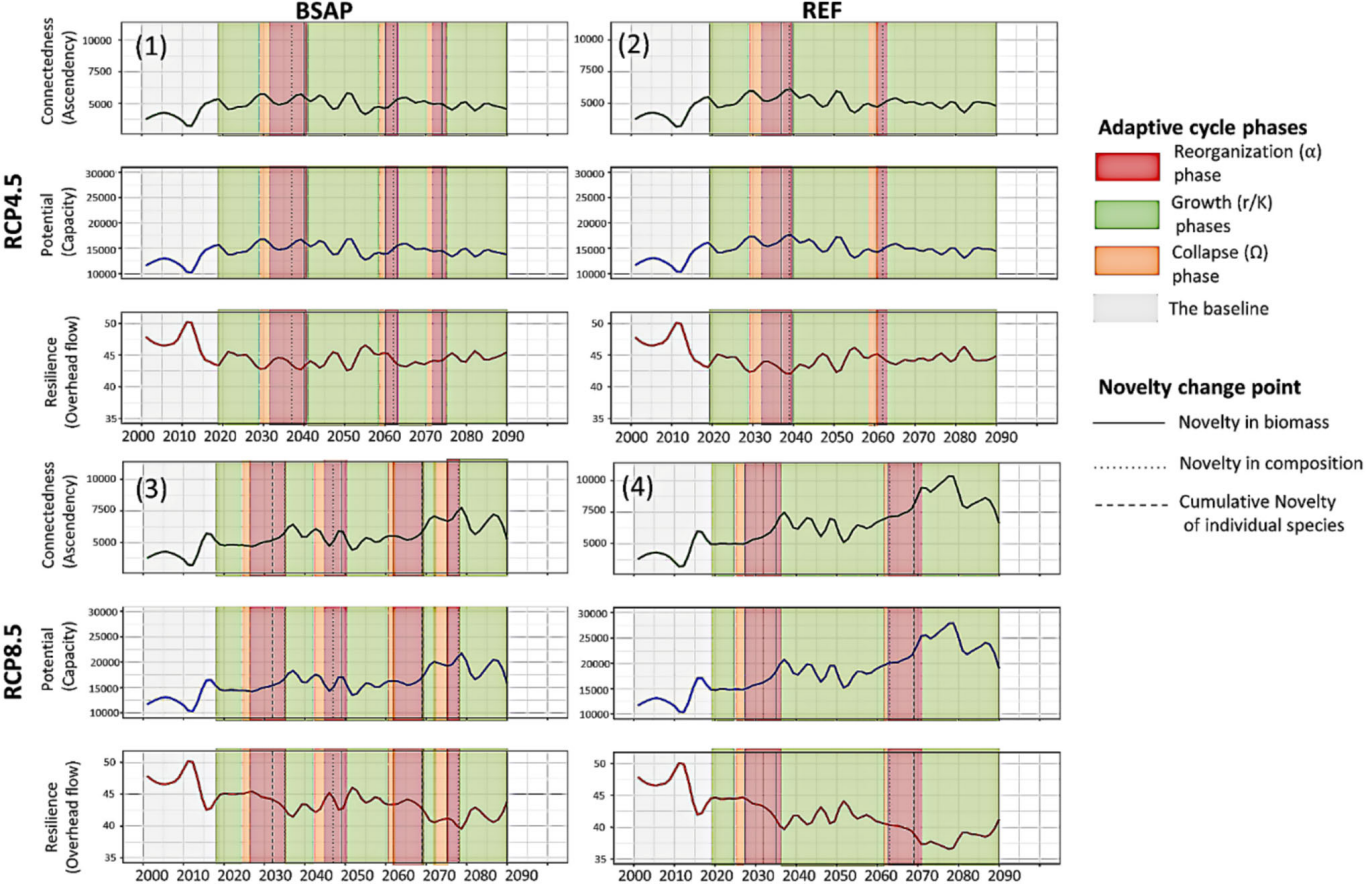
Ascendency, capacity, and overhead flow as respective indicators of the adaptive cycle dimensions connectedness, potential, and resilience for the four ecosystem trajectories identified in Fig. 3 (top left corner). The color bars indicate the different phases of the adaptive cycle color-coded as in Fig. 1. Different change points for novelty metrics time series indicate the reorganization phase. Three cycles were identified in trajectory 1 (RCP4.5_BSAP), two cycles in trajectory 2 (RCP4.5_REF), four cycles in trajectory 3 (RCP8.5_BSAP) and two cycles in trajectory 4 (RCP8.5_REF). The baseline 2000–2018 is shown in gray. All trajectories are under the SQ fishing pressure (See SupInfo Fig. S10 for other fishing pressure scenarios)

Using network indices capacity, ascendency, and overhead flow as respective indicators of potential, connectedness and resilience adaptive cycle dimensions, we identified the reorganization phases using significant change points in mean novelty in biomass and composition and qualitatively classified the collapse and growth phases for each trajectory (Fig. 6).

Our results suggest that trajectories emerging from low nutrient load management scenarios had more and faster reorganization phases than REF management scenarios (Fig. 6). The clear water trajectory 1 (RCP4.5_BSAP) showed three reorganization phases identified in the 2030s, 2060s, and 2070s (Fig. 6). Trajectory 3 (RCP8.5_BSAP) indicates four reorganization phases identified in the mid-2020s to 2030s, the second half of the 2040s, the 2060s, and the mid-2070s. However, both trajectories 2 (RCP4.5_REF) and 4 (RCP8.5_REF) show two reorganization phases, observed in the 2030s and early 2060s for trajectory 2 and in mid-2020s to 2030s and in 2060s for trajectory 4.

Under the same climate scenario, resilience was better maintained with the low nutrient load scenario (BSAP), which exhibited more cycles, compared to high nutrient load scenario (REF). In other words, decreasing nutrient load (BSAP) supported more frequent system reorganization, promoting greater resilience, whereas high nutrient load (REF) resulted in less system reorganization and lower resilience.

The duration of the reorganization phases differed within and between trajectories, with the first reorganization phase for each trajectory generally being the longest. The collapse phase was consistently shorter than the reorganization phase. The growth phase length was inconsistent within and between trajectories.

## Discussion

We quantified emergent novelty resulting from the compound effects of climate, nutrient load, and fishing and assessed its impact on FAS ecosystem resilience using the results of modeled future scenarios. Future trajectories were mainly shaped by the projected interaction of climate and nutrient load management scenarios. Beyond examining how novelty influences resilience, we delved deeper into ecosystem transitions through phases that can enhance or diminish its resilience. Using the adaptive cycle as an analytical framework, we analyzed how novelty and variability influence resilience. We found that resilience decreased with higher and faster novelty encountered under warmer climates (RCP8.5). Conversely, under low nutrient load scenarios (BSAP), the ecosystem reorganized faster, maintaining greater resilience regardless of climate conditions, compared to high nutrient load scenarios (REF). These findings highlight how climate change and anthropogenic pressures influence novelty magnitude and rate, shaping ecosystem resilience, in line with core resilience theory. Our analytical framework effectively captures novelty and adaptive cycle phases from model outputs, providing a tool to investigate resilience beyond the FAS ecosystem.

### Adaptive cycle: A tool for managing resilience

The conservation phase is often considered the most desirable for humans aiming to manage ecosystems, although it is the least resilient adaptive cycle phase (Fig. 1). To enhance ecosystem resilience, it is important to understand the transitions between phases and how these shifts influence overall resilience. The adaptive cycle offers a framework for understanding systems’ development from a baseline under certain conditions, and its deviations due to interactions between pressures and internal dynamics (Schrenk et al. 2022). In our study, the adaptive cycle uncovered the development and variability of the FAS from the baseline shaped by compound pressures and the emergence of novelty, influencing resilience. The relationship between novelty and resilience is complex as changes in the ecosystem structure and functions can vary. Our findings confirm that understanding ecosystems variability and how they generate novelty can enhance our understanding of resilience (Allen and Holling 2010). Such insight could inform on the windows of opportunities for management and highlight phases when most uncertainty and surprises can be expected.

The adaptive cycle phases across the four trajectories demonstrated that cycle frequencies vary with pressures. The reorganization of the FAS was more frequent under low nutrient load BSAP scenarios, with even faster cycles under the warmer climate scenario (RCP8.5_BSAP). In contrast, only two cycles were identified under REF nutrient load scenario, suggesting that higher nutrient loads might limit the ecosystem’s ability to undergo frequent reorganization. Furthermore, faster adaptive cycles under the BSAP scenarios contributed to maintaining resilience better than trajectories under REF scenarios for the same climate scenario. Hence, nutrient management could significantly enhance ecosystem resilience by facilitating quicker recovery and adaptation to environmental changes in the FAS ecosystem. This finding corroborates that management of local stressors could act against climate change effects to enhance ecosystem resilience (Scheffer et al. 2015).

The phase durations identified in this study deviate from the theoretical framework of the adaptive cycle where fast collapse and reorganization are followed by slower growth and conservation, supported by Castell and Schrenk (2020) and Schrenk et al. (2022). The duration of the phases differed even within the same trajectory. Hence, the emergence of novelty in the ecosystem under the compound anthropogenic pressures could increase irregularities of adaptive cycle frequencies and phase durations. Previous research has shown that environmental changes can alter the regularity of summer phytoplankton succession (Angeler et al. 2015). At the ecosystem level, irregularities may lead to deceptive interpretations and maladaptive responses in adaptive management cyclic interventions (e.g., BSAP, fisheries management). One maladaptive response could be to reduce the system variability, which is risky as it can obscure signals of decreasing resilience and suppress the information needed for effective adaptive management, ultimately undermining the adaptive capacity that builds stress tolerance (Carpenter et al. 2015). For example, rigid management of the Baltic Sea fisheries (single-species stable quotas) has suppressed variability and fishers’ flexibility to adapt to environmental changes, jeopardizing resilience (Ammar et al. 2021a). We highlight the need to advance the understanding of adaptive cycle phase durations and irregularities under different pressures to prevent unintended management outcomes such as not achieving a Good Environmental Status.

### FAS current state, future novelty, and resilience

The current environmental state of the FAS coastal ecosystem (baseline period) has raised significant concerns (HELCOM 2023). Despite nutrient loads reductions before and during the baseline period, the FAS state has not followed the same improvement patterns across the entire Baltic. In the model projections, a Good Environmental Status as defined by the Marine Strategy Framework Directive (desirable state) was only achieved in the RCP4.5 climate scenario adhering to BSAP. Notably, nutrient reductions and fisheries management scenarios may prove insufficient to reach a Good Environmental Status if climate change follows the RCP8.5 climate trajectory, emphasizing the need for climate-adaptive management to sustain resilience.

In the early twentieth century, the FAS ecosystem had abundant *Fucus* when the water transparency was around 8–10 m in the adjacent areas (Fleming-Lehtinen and Laamanen 2012). Although clear water states have existed in the past, it has never been in conjunction with higher temperatures, and the current mix of native (e.g., sander and perch) and non-indigenous (e.g., round goby, Harris mud crab, and fish-hook water flea) species. In this context, the emerging clear water system in trajectory 1 (RCP4.5_BSAP) is considered novel compared to past clear water states. Trajectories 2 and 3 are the least novel (the closest to the baseline), although they are novel compared to historical clear water states in the FAS. Ecosystems exhibit context-dependent identities, meaning the same starting conditions can lead to different trajectories, making baselines subjective and influenced by human perceptions of change, known as the shifting baseline (Pauly 1995). While some suggest defining baselines based on minimal human impact (Rodrigues et al. 2019), long-term studies indicate that novel states can persist (Pandolfi et al. 2020), challenging fixed historical baselines for conservation. In this study, we focus on trajectories emerging from the current-state baseline, possible future novelty and resilience opportunities.

Novelty in biomass reflects system-level changes driven by shifts in species composition and ecological processes, while novelty in composition reflects adaptive capacity through adjustments of species dominance in response to stressors (Dornelas and Madin 2020; Pandolfi et al. 2020). Our results demonstrate that while high levels of change can generate novelty, this does not necessarily lead to reducing resilience. The highest novelty in biomass was identified in trajectory 4 (RCP8.5_REF) followed by trajectory 3 (RCP8.5_BSAP) where the more intense climate change affects all species biomasses and contributes to decreasing resilience. However, the highest novelty in composition occurred in trajectory 1 (RCP4.5_BSAP), followed by trajectory 4 (RCP8.5_REF). In this case, species composition varies as a response to the eutrophication status, where eutrophic waters and climate warming promote filamentous algae, contributing to decreasing resilience. The decline in biomass and proportion of the macroalga *Fucus vesiculosus,* a key species in the FAS and Baltic coastal communities, also contributes to decreasing resilience. Interestingly, invasive species increase did not appear to affect resilience across trajectories. Hence, resilience declined under eutrophication, which showed high novelty in biomass and composition, reflecting species loss. In contrast, trajectory 1 maintained resilience despite compositional changes, suggesting the system adapted by sustaining functionally similar species and preserving ecosystem functions, which aligns with core resilience theory.

Although resilient states are often viewed positively, they are not necessarily desirable. For instance, trajectory 2 (RCP4.5_REF) is resilient compared to the RCP8.5 trajectories, but may not be desirable. Additionally, novelty from invasive species might have fostered adaptation contributing to resilience, although usually considered undesirable. This highlights the need to understand the compound impacts of local to global scale stressors on ecosystems and different novelty characteristics (e.g., biomass and composition) to understand resilience.

### Novelty and reorganization

When a system is disrupted, it can reorganize by altering the dominance of existing elements, or through *bricolage* which refers to the recombination of old and new elements (Gunderson and Holling 2002). Reorganization thus requires either a novel structure of the existing elements or a novel combination of existing and new ones (Gunderson and Holling 2002). In this study, species represent the FAS ecosystem model elements. However, models are a simplification of nature with low opportunities for bricolage (e.g., no introduction of new species or functions). Changes in real ecosystems, especially with extreme events not captured in models, are often greater. Hence, novelty in composition in this study (maximum 0.16 in [0, √2] range) is likely low compared to what could be expected in natural ecosystems arising from higher turnovers, which in turn could lead to further resilience loss, opportunities for bricolage, and irregularities in adaptive cycle phases. The significance of these results therefore lies in the differences between scenarios that illustrate general trajectories rather than absolute future biomasses in the ecosystem.

Ecosystem studies indicate that the rate of change can be more important than specific ecosystem states (Dornelas and Madin 2020; Williams et al. 2021). Faster warming (RCP8.5) scenarios triggered higher and faster novelty causing a resilience decline. The current anthropogenic climate change rate has not been experienced previously on the Earth System and might generate further species turnover (Burke et al. 2018; Jonkers et al. 2019). This can lead to high novelty in species composition coupled with a high extinction rate as novel climate conditions may not match the adaptive capacity of some species (Dornelas and Madin 2020; Williams et al. 2021; Penn and Deutsch 2022), especially those from colder regions such as FAS. Additionally, physiological optima and environmental tolerance of species within a community can differ and result in shifts in the strength and outcome of species interactions (Kroeker and Sanford 2022). Consequently, there is a need to prevent fast emergence of novelty in species assemblages, associated with fast climate change and anthropogenic pressures.

### Outlook

Models are useful tools for understanding (eco)systems dynamics and developing theories despite limitations (e.g., ignoring stochastic changes). Here, we focused on adaptive cycles, novelty and resilience using the FAS ecosystem model and future scenarios. The network indices derived from Ecopath with Ecosim models offers opportunities to use adaptive cycle framework as a tool to investigate resilience-related questions beyond the FAS ecosystem. Such additions could improve the understanding of resilience and reduce uncertainties in adaptive management interventions under unpredictable future events.

Adaptive cycles at different levels (e.g., socioeconomic, ecosystem services, and their interactions) and scales (e.g., spatial and temporal) are important to account for in management. For instance, fisheries management interventions at different governance levels can affect the whole social-ecological system (Ammar et al. 2021a). Accounting for the emergence of novelty at different spatiotemporal scales and social-ecological levels is important to understand system resilience. Applying this paper’s method to a nested set of adaptive cycles (Gunderson and Holling 2002) could help improve our knowledge of variability across scales and the type of novelty that could promote resilience and desirable states.

## Supplementary Information

Below is the link to the electronic supplementary material.

Supplementary file1 (PDF 2121 kb)

## Data Availability

Due to their size, EwE data are not publicly shared but are available upon request with the developer’s permission.

## Footnotes

1 Complex adaptive systems (CAS) are systems that exhibit nonlinear behavior, display multiple states, and have the capacity to adapt, evolve, and learn (Holland 1992; Levin 1998; Gunderson and Holling 2002, Chaffin et al. 2016, Garmestani et al. 2020).

2 https://ecopath.org/.
