# Supplementary material for: Novelty, variability, and resilience: Exploring adaptive cycles in a marine ecosystem under pressure
Source: Ambio. 2025 Apr 22;54(11):1885–901. doi: 10.1007/s13280-025-02181-1 (PMC12480343; doi:10.1007/s13280-025-02181-1)
Supplement: Supplementary file 1 — Supplementary file1 (PDF 2121 kb) [file 13280_2025_2181_MOESM1_ESM.pdf]

Ambio

Supplementary Information

This supplementary information has not been peer reviewed.

Title: **Novelty, variability, and resilience: exploring adaptive cycles in a marine ecosystem under pressure.**

Yosr $Ammar^{1,2*}$, Riikka $Puntila-Dodd^{3,4}$, Maciej T. $Tomczak^{5}$, Magnus $Nyström^{1}$, Thorsten $Blenckner^{1}$

[1] Stockholm Resilience Centre, Stockholm University, Stockholm, Sweden

[2] Department of Environmental Research and Monitoring, Swedish Museum of Natural History, Stockholm, Sweden

[3] Marine and Freshwater solutions, Finnish Environment Institute, Helsinki, Finland

[4] Department of Environmental and Marine Biology, Åbo Akademi University, Åbo, Finland

[5] Department of Aquatic Resources (SLU aqua) at the Swedish University of Agricultural Sciences

1. ***Foodweb structure***
2. ***EwE model forcing, calibration and uncertainty simulations***
3. ***Ecosystem trajectories and food web***
4. ***Novelty, change points, and resilience***
5. ***The adaptive cycle phases***

1. Food web structure

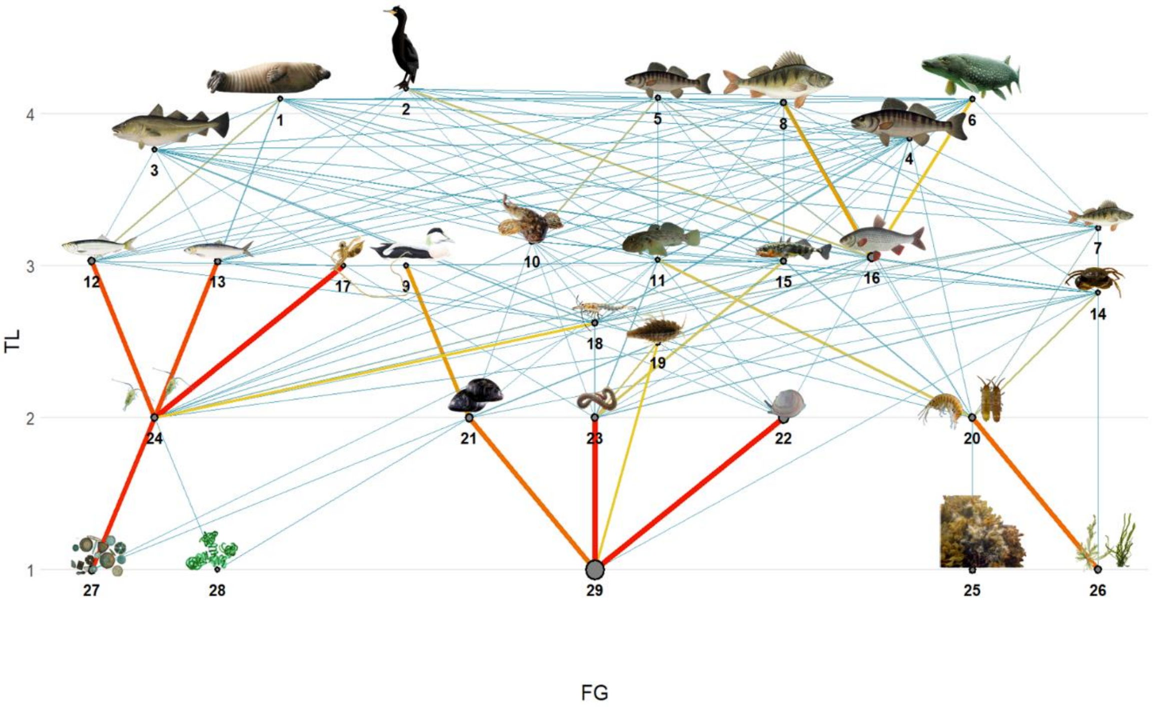

Figure.S 1: Biological groups and their trophic relationships in the Finnish Archipelago Sea the Ecopath with Ecosim model.

2. EwE model forcing, calibration and uncertainty simulations

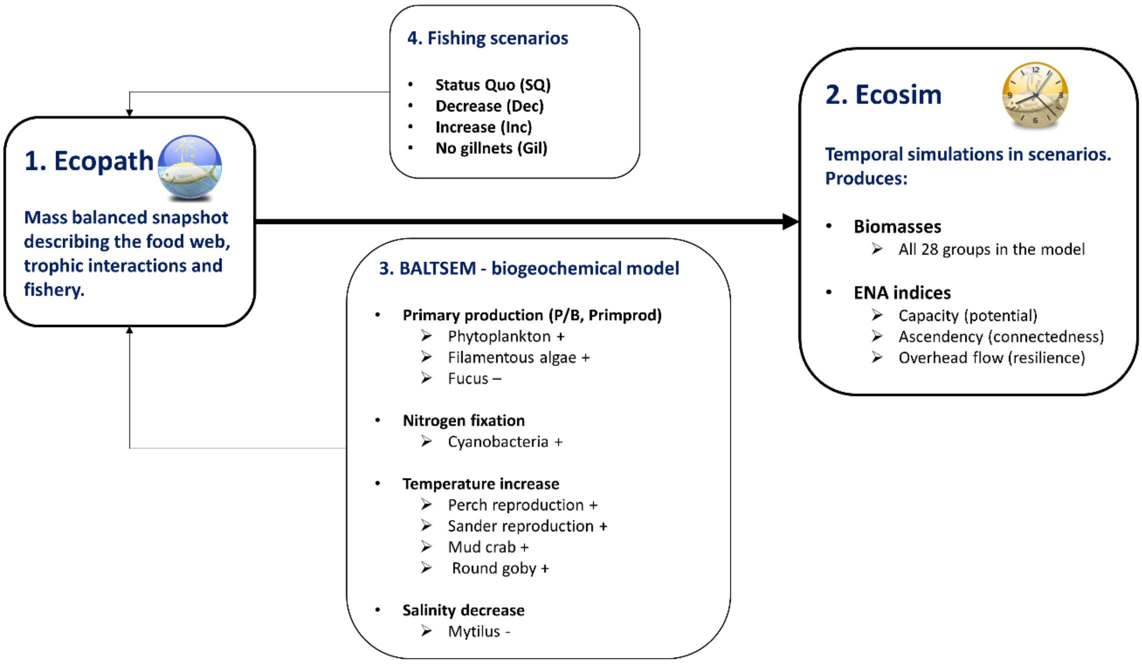

Figure.S 2: Ecopath with Ecosim model and scenarios building process for the Finnish Archipelago Sea. The full model description is given in Puntila-Dodd et al. (2022).

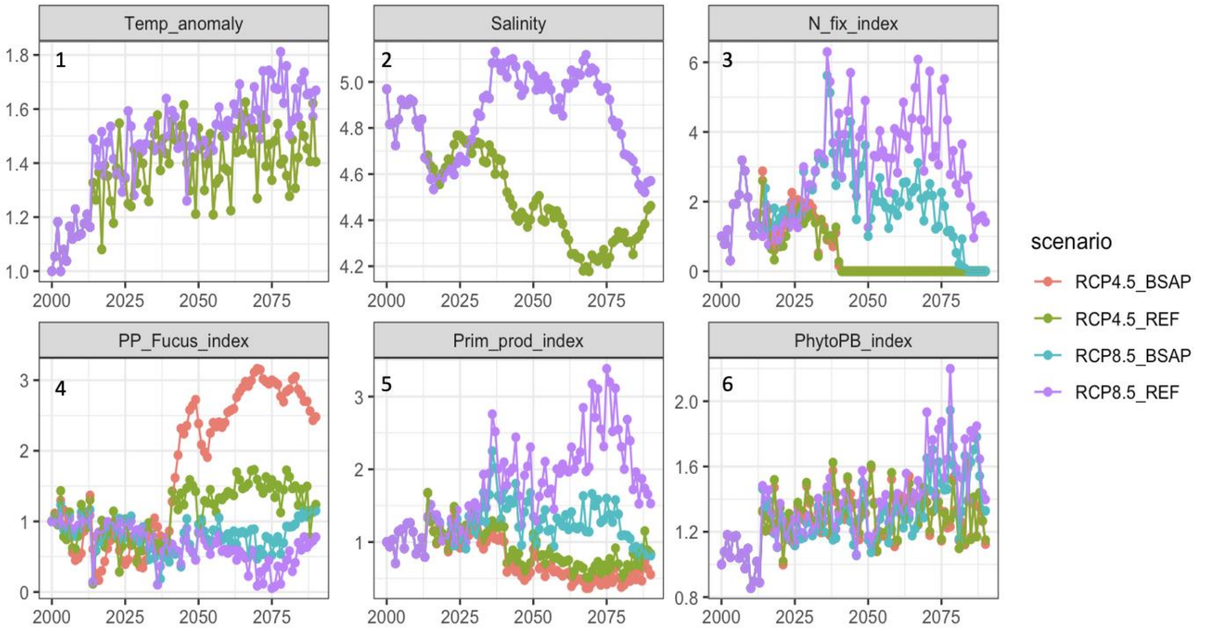

Figure.S 3: Preview of differences between the compound of climate and nutrient load scenarios in 1) temperature anomaly 2) salinity, 3) Nitrogen fixation index, 4) primary production by Fucus index, 5) primary production, 6) and phytoplankton production biomass index.

Environmental forcing for the food-web model, as well as the projections for nutrient and chlorophyll-a concentration, were based on climate and eutrophication scenarios generated by the biogeochemical BALTSEM model (Fig.S2-3). BALTSEM describes the dynamics of nitrate, ammonium, phosphate, three phytoplankton taxa, zooplankton, detritus, and oxygen (Eilola et al. 2009; Gustafsson et al. 2012; Savchuk et al. 2012) as a set of 13 horizontally homogeneous coupled Baltic Sea basins with high vertical resolution. Climate change and biogeochemical scenarios were run with atmospheric forcing based on three downscaled global General Circulation Models (GCMs), the Max Planck Institute Earth System Model-Low Resolution (MPI-ESM-LR), the European Countries Earth System Model (EC-EARTH), the Hadley Center Global Environment Model version 2 - Earth System (HadGEM2-ES), and later models A, B, D (see for details (Saraiva et al. 2019)). GCM output was downscaled by the coupled Rossby Center Atmosphere Version 4 and Nucleus for European Modeling of the Ocean model (RCA4-NEMO, for details, see Saraiva et al. 2019).

The EwE Ecosampler was applied to estimate the uncertainty of the basic parameters and assumptions in the EwE model (Steenbeek et al. 2018). It employs a Monte Carlo (MC) routine, applying alternate parameter sets that result a mass-balanced model to measure the impact of input parameter sensitivity on the model's results (Steenbeek et al. 2018). A number of 400 MC runs were conducted for each scenario providing a set of temporal results with uncertainty around the median for all functional groups and ENA indices. Further description and uncertainty simulations are described in the SupInfo (paragraph 2) and Puntila-Dodd et al., (2022).

An EwE-wide uncertainty assessment module varies the Ecopath initial condition input parameter set (Biomass, Production per Biomass, Consumption per Biomass, Catches, and Diet composition). The coefficient of variation was obtained from data pedigree, i.e., a measure of confidence in the data source of each parameter given by the modelers (ICES 2016). MC then resamples alternate mass-balanced models, where the entire energy in the system is accounted for without requiring outside sinks or sources (ecotropic efficiency values <= 1) with uncertainty around the median.

3. Ecosystem trajectories and food web

To understand the trajectories of the food-web (biomasses of all groups) under different scenarios and the resulting ecosystem states, we identify the number of trajectories of the food-web over time. The different trajectories were analyzed using the mclust package (Scrucca et al. 2016), which builds on model-based clustering. The model-based clustering in turn is predicted on parameterized finite Gaussian mixture models which select the optimal models from an expectation–maximization algorithm initialized by hierarchical

model-based agglomerative clustering (Scrucca et al. 2016). This package allowed the extraction of a number of trajectories across the scenarios. A continuously evolving trajectory represents a system direction influenced and maintained by endogenous self-reinforcing processes.

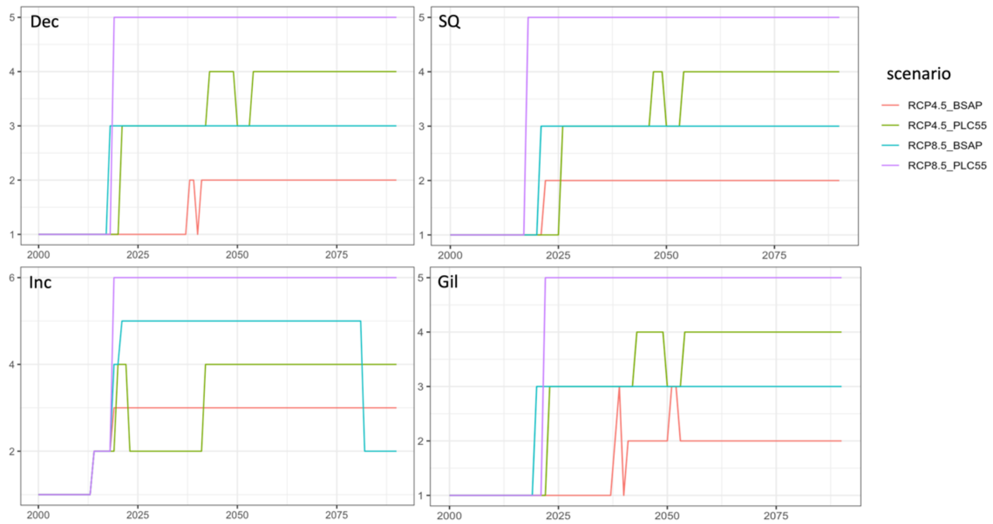

Figure.S 4: Number of clusters under the compound effects of climate and nutrient load management scenarios for the four fishing management scenarios used to identify and understand trajectories

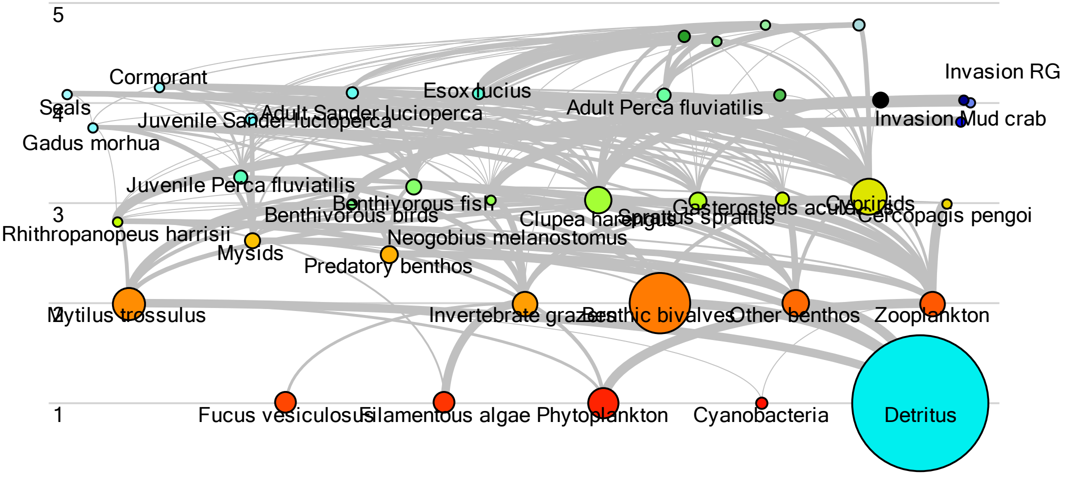

Figure.S 5: Baseline (2000-2018) state of biomass and flows between species/groups.

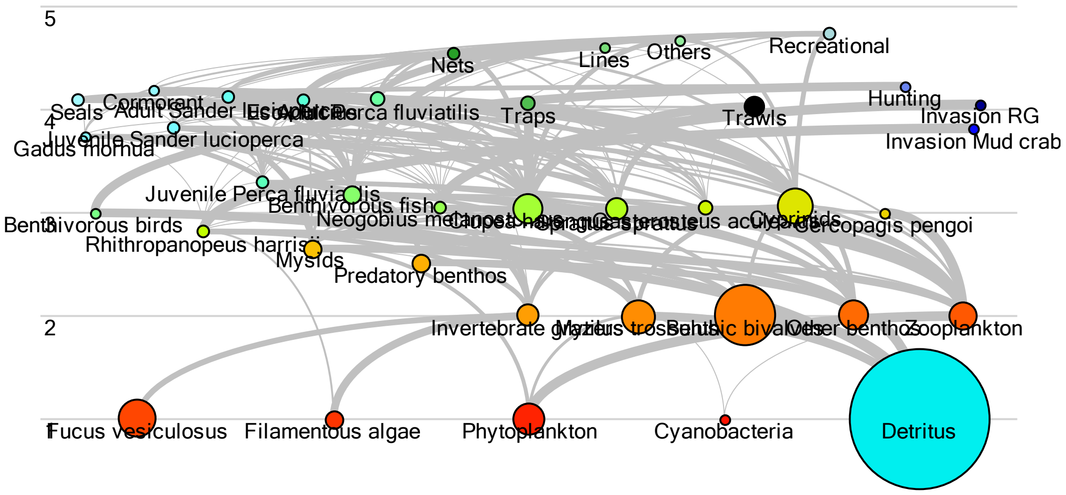

Figure.S 6a: RCP4.5_BSAP of biomass and flows between species/groups in 2050.

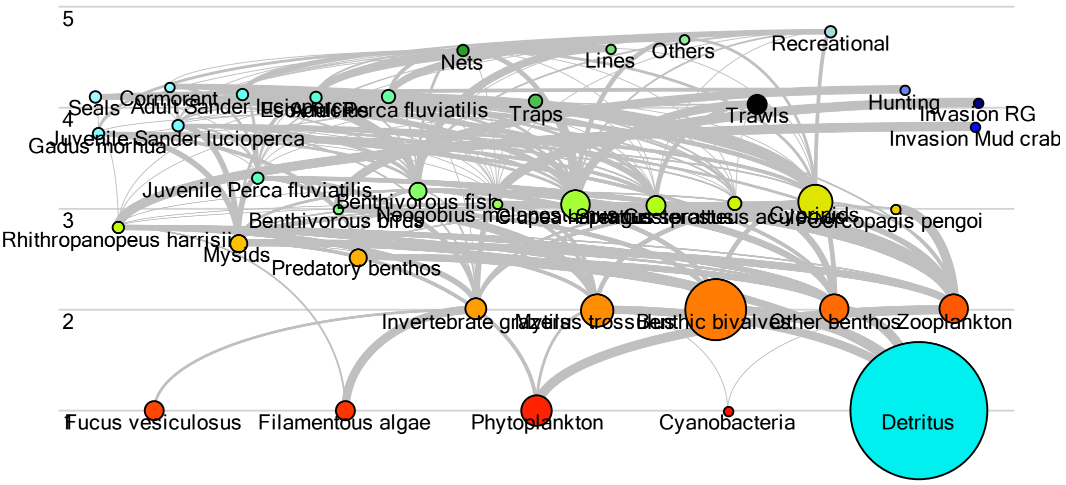

Figure.S. 6b: RCP4.5_REF of biomass and flows between species/groups in 2050.

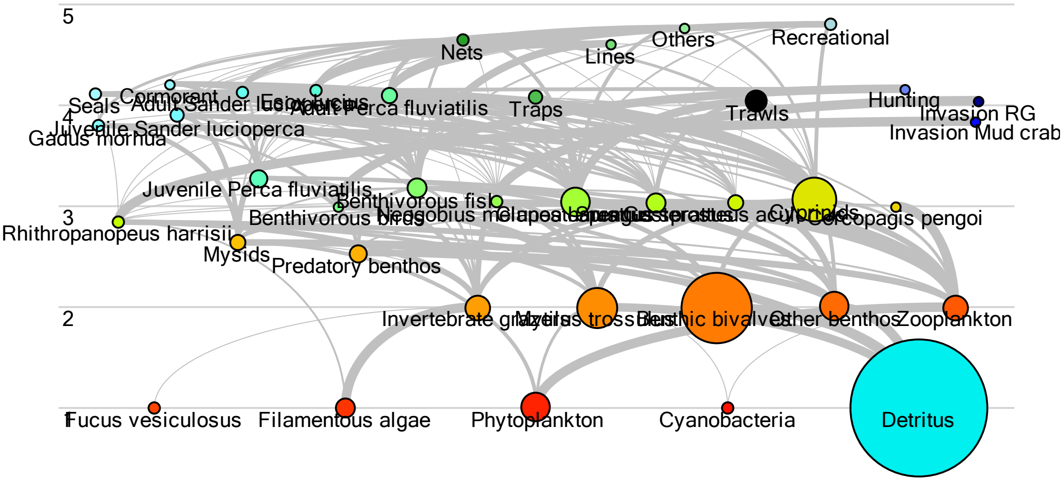

Figure.S.6c: RCP8.5_BSAP of biomass and flows between species/groups in 2050.

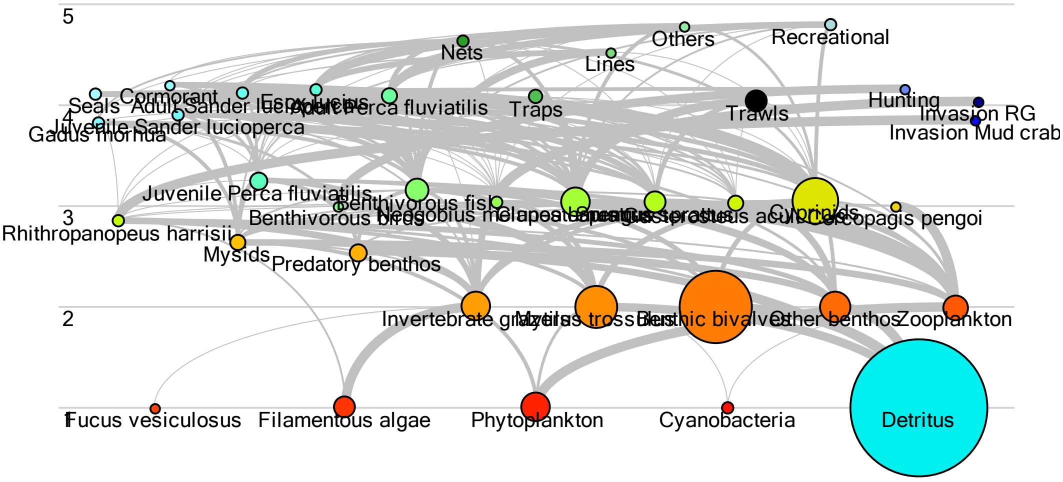

Figure.S.6d: RCP8.5_REF of biomass and flows between species/groups in 2050.

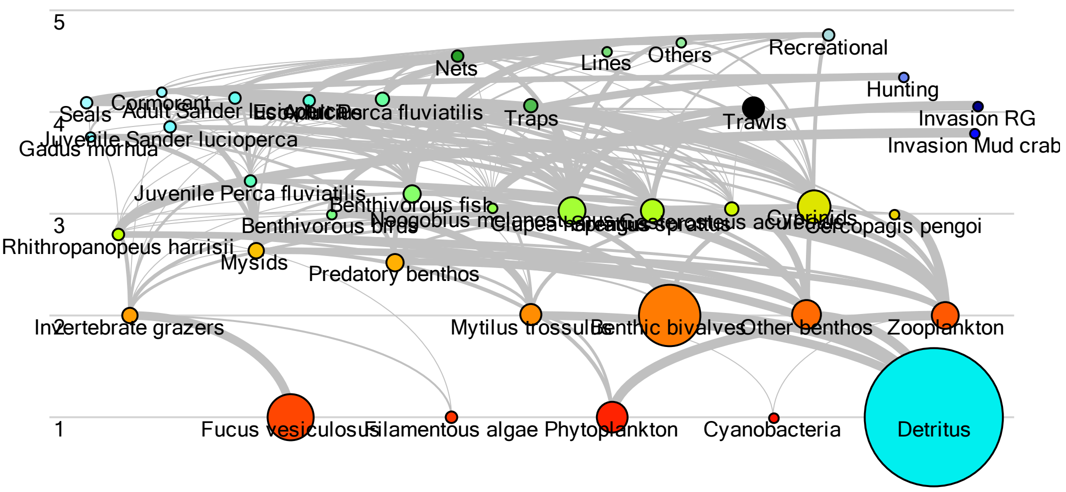

Figure.S 7a: RCP4.5_BSAP of biomass and flows between species/groups in 2070.

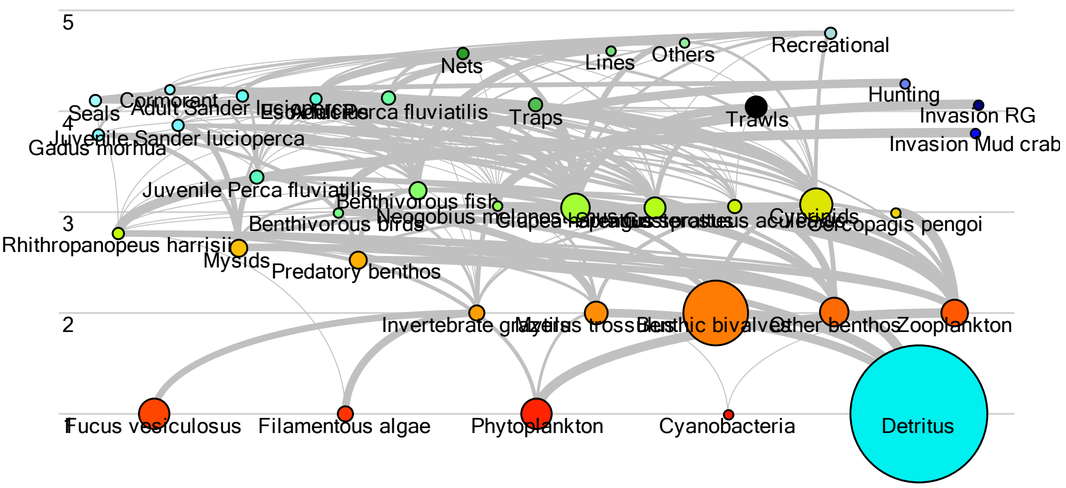

Figure.S. 7b: RCP4.5_REF of biomass and flows between species/groups in 2070.

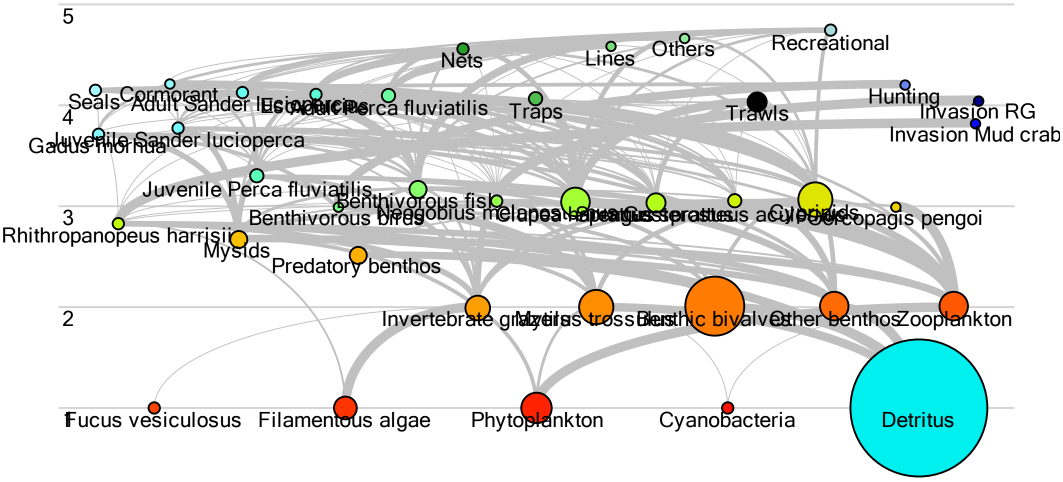

Figure.S.7c: RCP8.5_BSAP of biomass and flows between species/groups in 2070.

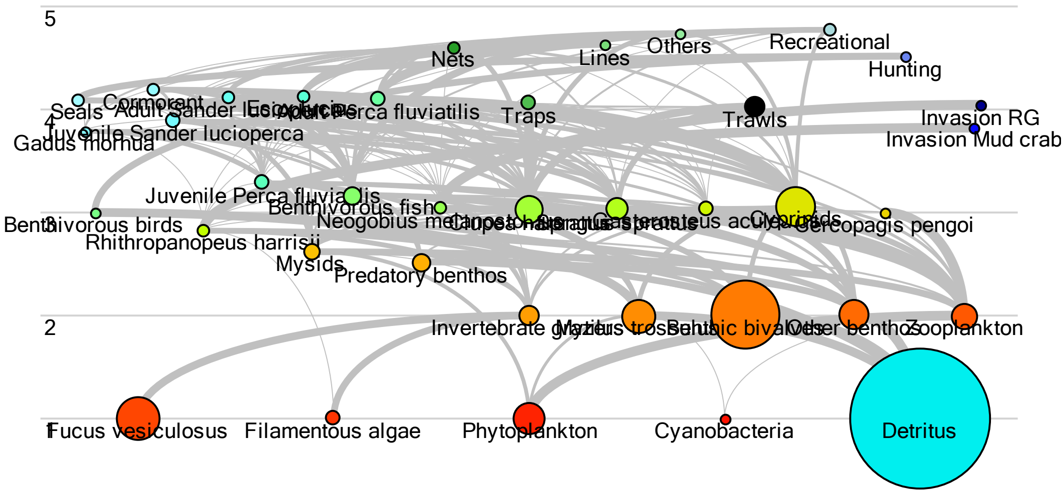

Figure.S.7d: RCP4.5_BSAP of biomass and flows between species/groups in 2090.

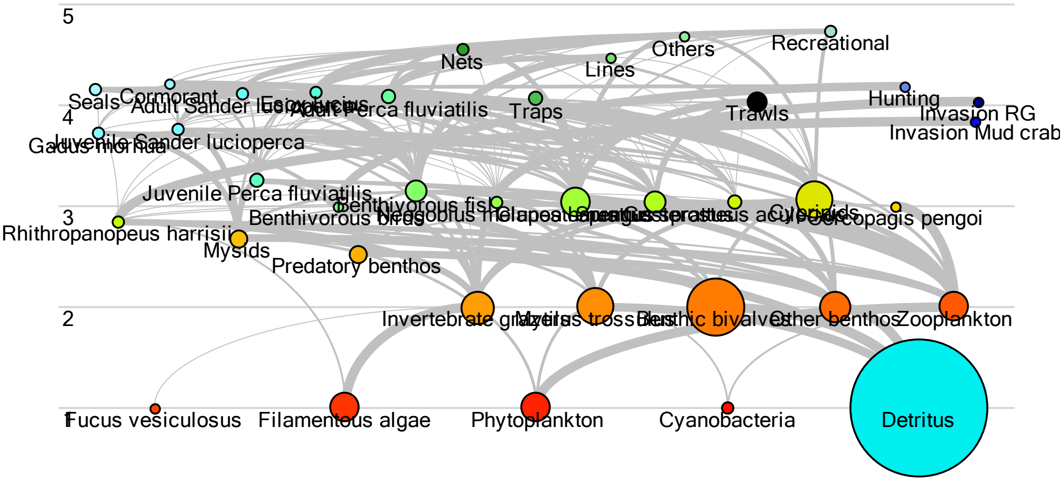

Figure.S 8a: RCP8.5_REF of biomass and flows between species/groups in 2070.

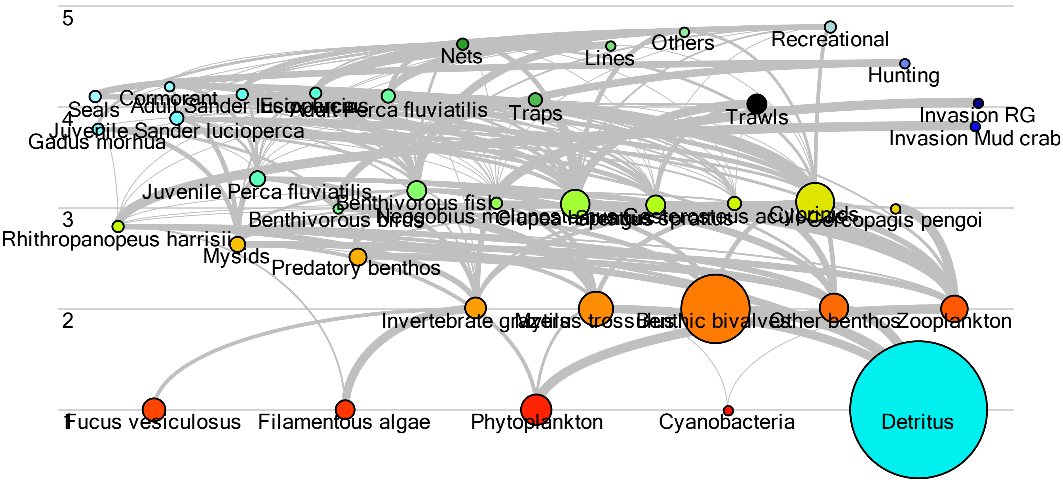

Figure.S. 8b: RCP4.5_REF of biomass and flows between species/groups in 2090.

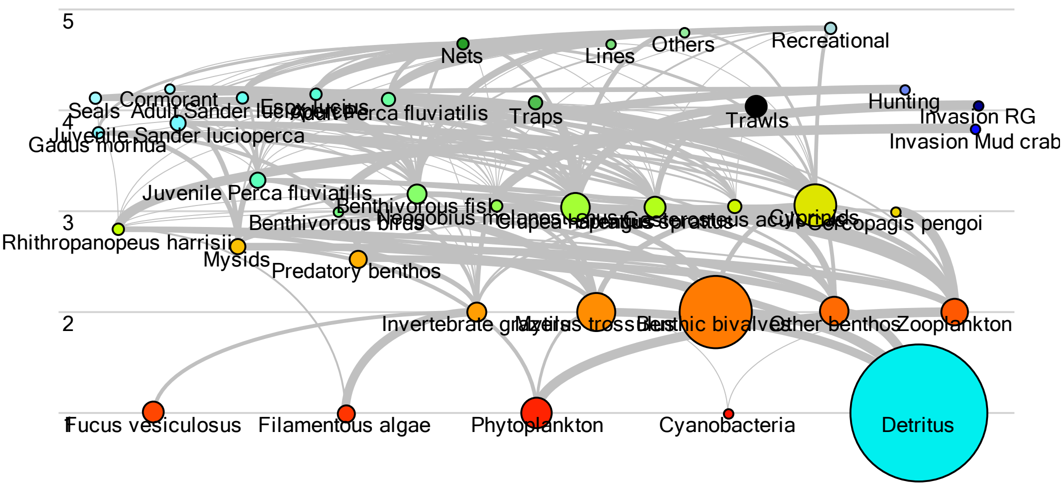

Figure.S.8c: RCP8.5_BSAP of biomass and flows between species/groups in 2090.

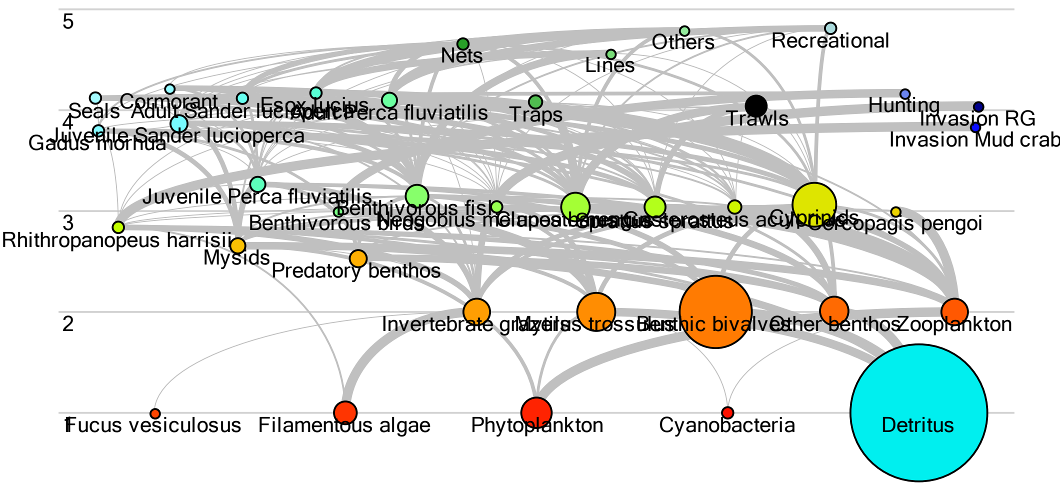

Figure.S.8d: RCP8.5_REF of biomass and flows between species/groups in 2090.

4. Novelty, change points, and resilience

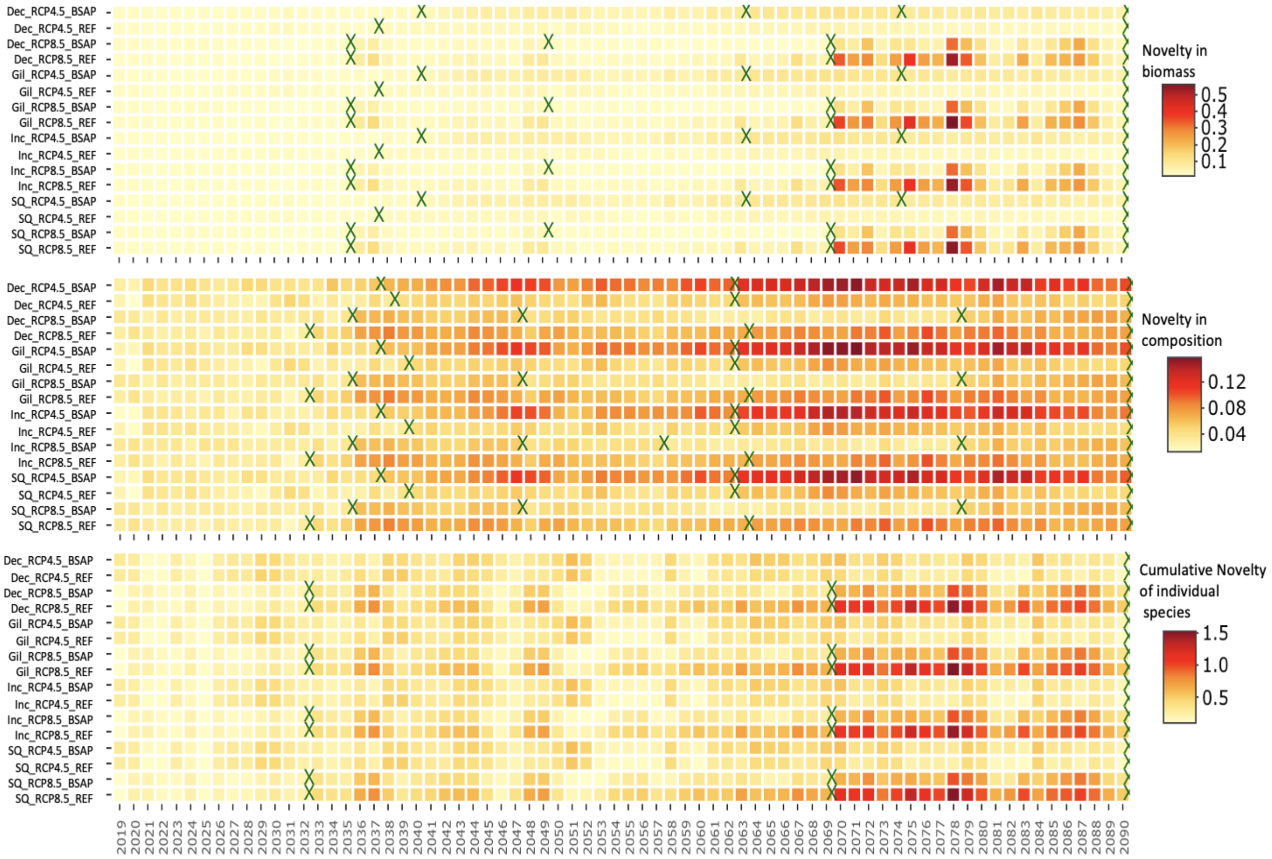

Figure.S 9: Novelty in biomass, composition, and cumulative novelty of individual species compared to the 2000-2018 baseline in the Archipelago Sea. The detection of change points is point marked by the green (X).

The novelty calculations resulted in distinct patterns across the different future trajectories. Overall, the nutrient and climate scenarios have a strong influence on novelty in composition, while the climate scenarios have an even stronger influence on both the biomass and cumulative novelty. Interestingly, the contribution of fishing management scenarios on novelty is low, and do not differ much across the fishing scenarios. The highest novelty relative to the baseline period is observed for the RCP8.5 trajectories, except for novelty in species composition. The RCP8.5 scenarios have a strong contribution to the emergence of novelty in biomass and cumulative novelty, especially after the 2070s. This is even larger with the REF nutrient scenario. The lowest novelty in biomass is observed for the RCP4.5_REF scenarios. The cumulative novelty follows similar trajectories to that of novelty in biomass, but with larger fluctuations.
Novelty in composition for RCP4.5_BSAP is high, starting from the 2040s increasing towards the late 2060s and 2070s. Following that, both climate scenarios with REF show high novelty in composition for the same periods, with a lower amplitude than the RCP8.5_BSAP scenario. The lowest novelty in composition is observed in the RCP4.5_REF scenarios.
The change point analyses indicate that novelty in composition, biomass, and cumulative novelty of individual species may occur consecutively (Fig.S9). For instance, in the RCP4.5_BSAP scenarios, change points in composition at two time periods are found before that of the biomass, while no significant change point is detected in cumulative novelty of individual species. In general, change points are detected in novelty in composition (and cumulative novelty of individual species) around the 2030s, followed by change points in novelty in biomass in most scenarios. There are also change points in novelty in composition followed by novelty in biomass in the RCP8.5_BSAP. Change points in novelty in composition are detected around the 2060s followed by biomass and cumulative novelty, and change points in novelty occur in composition in the late 2070s. Finally, the lowest number of change points is found for the cumulative novelty.

5. The adaptive cycle phases

The adaptive cycle phases vary within the three axes: connectedness, potential and resilience (Table S1). The rapid growth and accumulation of capital phase (r) followed by the slow growth and conservation phase (K) make the system increasingly overconnected, thus, losing its resilience. This fosters the collapse phase (Ω), essential to offer conditions of low connectedness and higher resilience and potential for the (α) reorganization phase of novelty and experiment (Gunderson and Holling 2002). The low connectedness allows novel reassortments of elements that previously were tightly connected while high resilience allows experimenting with these novel combinations (Gunderson and Holling 2002). The reorganization phase is characterized by great uncertainty, change of unexpected forms (novelty), and unexpected crises, but provides great potential for a new adaptive cycle (Gunderson and Holling 2002).

Table S 1: Trends in the adaptive cycle phases of the three axes: connectedness, potential and resilience as described in Gunderson & Holling, (2002). In the rapid and slow growth (r) and (K) phases, the system connectedness and potential increase while resilience decrease. In the collapse phase connectedness decreases, resilience increases and potential decrease then increase. In the reorganization phase of novelty and experiment, resilience and potential are high while connectedness is low.

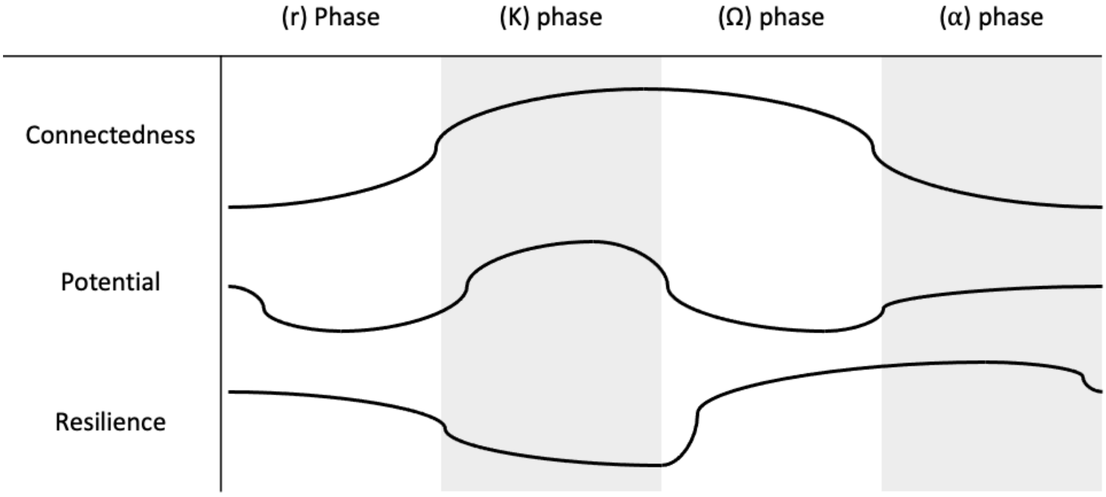

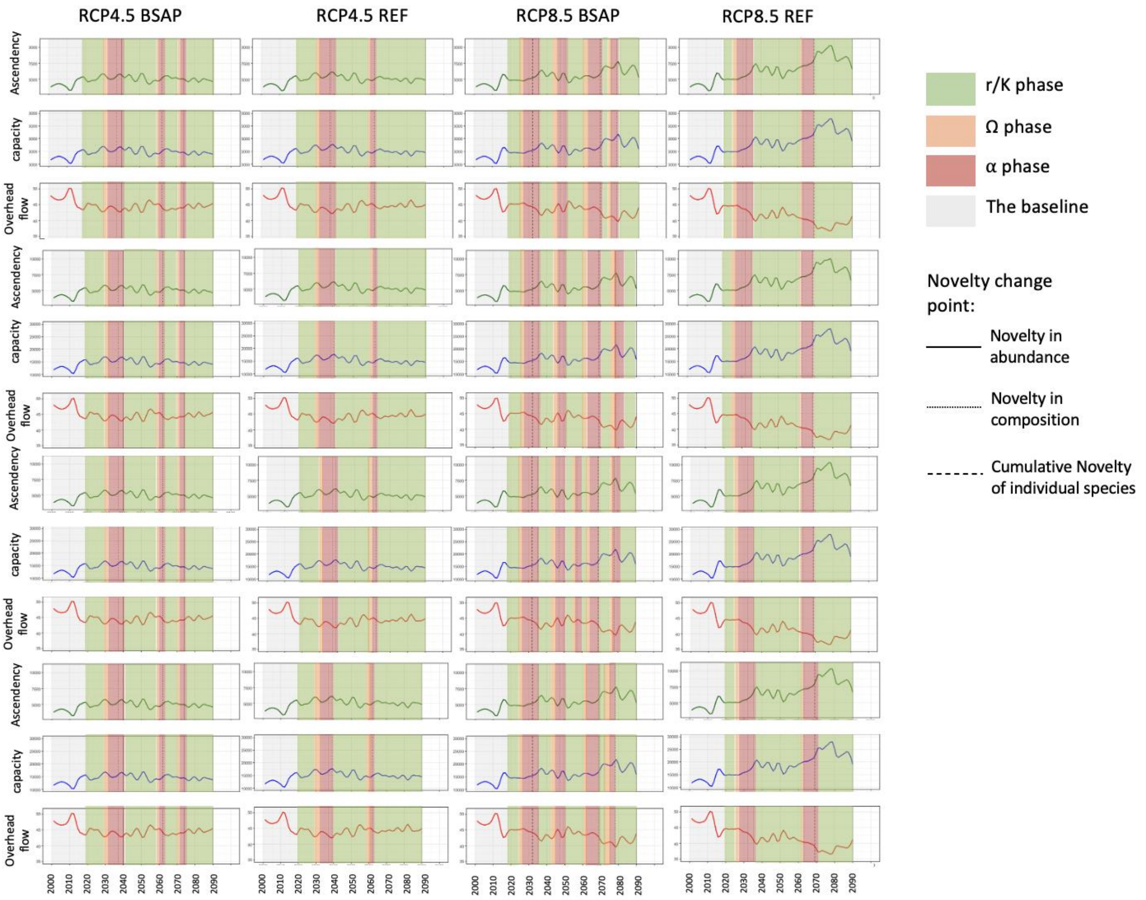

Figure.S 10: Ascendency, capacity and overhead flow indicators of connectedness, potential and resilience axes of the adaptive cycle respectively, in different scenarios of the Archipelago Sea. The colors indicate metaphorically the phases of the adaptive cycle. The baseline 2000-2018 is shown in grey. The different novelty metrics indicate the reorganization phase.

6. Novelty and resilience

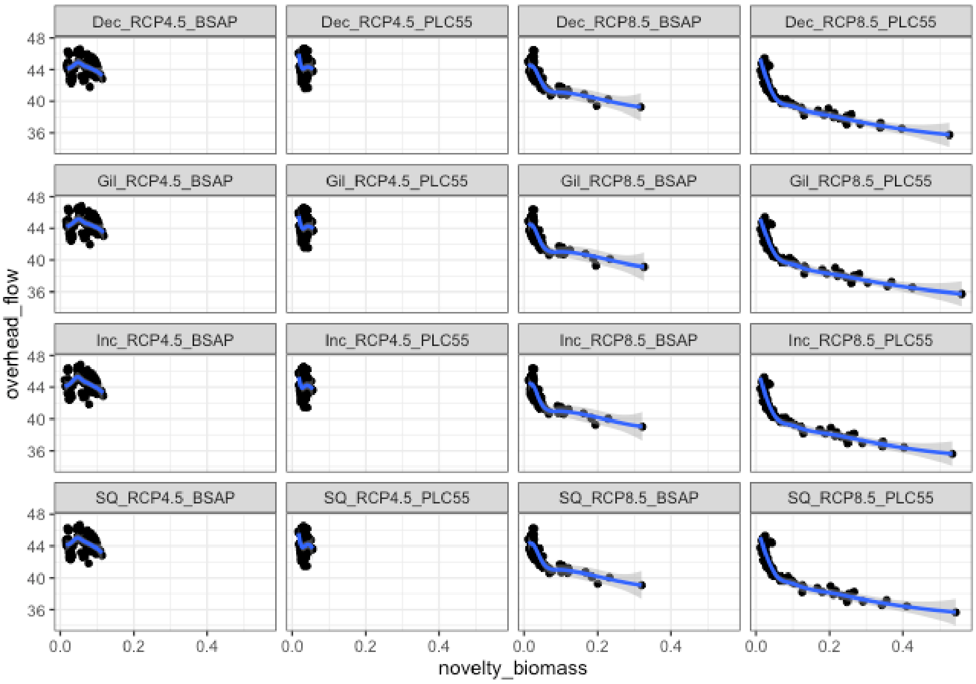

Figure.S 11: Novelty in biomass vs the Resilience indicator, overhead flow, in all scenarios

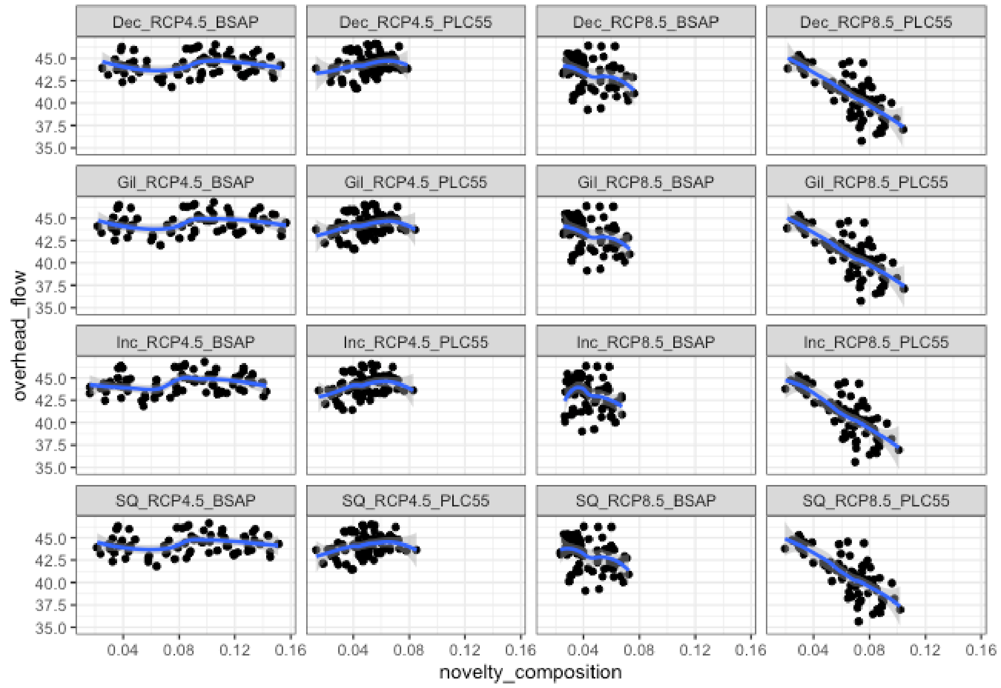

Figure.S 12: Novelty in composition vs the Resilience indicator, overhead flow, in all scenarios.

References

Eilola, K., H. M. Meier, and E. Almroth. 2009. On the dynamics of oxygen, phosphorus and cyanobacteria in the Baltic Sea; A model study. *Journal of Marine Systems* 75: 163–184.

Gunderson, L. H., and C. S. Holling, ed. 2002. *Panarchy: understanding transformations in human and natural systems*. Washington, DC: Island Press.

Gustafsson, B. G., F. Schenk, T. Blenckner, K. Eilola, H. E. M. Meier, B. Müller-Karulis, T. Neumann, T. Ruoho-Airola, et al. 2012. Reconstructing the Development of Baltic Sea Eutrophication 1850–2006. *AMBIO* 41: 534–548. doi:10.1007/s13280-012-0318-x.

ICES. 2016. *Report of the Working Group on Multispecies Assessment Methods (WGSAM)*. ICES CM 2016/SSGEPI 21. Reykjavik, Iceland.

Puntila-Dodd, R., H. Peltonen, O. Heikinheimo, J. Riitakorpi, B. Muller-Karulis, S. Niiranen, M. T. Tomczak, and L. Uusitalo. 2022. Combined effects of eutrophication, fishery and species introductions in a temperate coastal ecosystem: Modelling changes in the Archipelago Sea food web 2000-2016. In . Donostia – San Sebastian.

Saraiva, S., H. E. M. Meier, H. Andersson, A. Höglund, C. Dieterich, M. Gröger, R. Hordoir, and K. Eilola. 2019. Uncertainties in Projections of the Baltic Sea Ecosystem Driven by an Ensemble of Global Climate Models. *Frontiers in Earth Science* 6: 244. doi:10.3389/feart.2018.00244.

Savchuk, O. P., B. G. Gustafsson, and B. Muller-Karulis. 2012. *BALTSEM - a marine model for decision support within the Baltic Sea region*. Technical Report 7. Sweden: the Baltic Nest Institut.

Scrucca, L., M. Fop, T. Murphy Brendan, and A. Raftery E. 2016. mclust 5: Clustering, Classification and Density Estimation Using Gaussian Finite Mixture Models. *The R Journal* 8: 289. doi:10.32614/RJ-2016-021.

Steenbeek, J., X. Corrales, M. Platts, and M. Coll. 2018. Ecosampler: A new approach to assessing parameter uncertainty in Ecopath with Ecosim. *SoftwareX* 7: 198–204. doi:10.1016/j.softx.2018.06.004.
